# Tau fibrils evade autophagy by excessive p62 coating and TAX1BP1 exclusion

**DOI:** 10.1126/sciadv.adm8449

**Published:** 2024-06-12

**Authors:** Luca Ferrari, Bernd Bauer, Yue Qiu, Martina Schuschnig, Sigrid Klotz, Dorothea Anrather, Thomas Juretschke, Petra Beli, Ellen Gelpi, Sascha Martens

**Affiliations:** ^1^ Max Perutz Labs, Vienna Biocenter Campus (VBC), Dr.-Bohr-Gasse 9, 1030 Vienna, Austria.; ^2^ University of Vienna, Max Perutz Labs, Department of Biochemistry and Cell Biology, Dr.-Bohr-Gasse 9, 1030 Vienna, Austria.; ^3^ Vienna BioCenter PhD Program, Doctoral School of the University of Vienna and Medical University of Vienna, Campus-Vienna-Biocenter 1, 1030 Vienna, Austria.; ^4^ Division of Neuropathology and Neurochemistry, Department of Neurology, Medical University of Vienna, 1090 Vienna, Austria.; ^5^ Max Perutz Labs, Mass Spectrometry Facility, Vienna Biocenter Campus (VBC), Dr.-Bohr-Gasse 9, 1030 Vienna, Austria.; ^6^ Institute of Molecular Biology, 55128 Mainz, Germany.; ^7^ Institute of Developmental Biology and Neurobiology (IDN), Johannes Gutenberg-Universität, 55128 Mainz, Germany.

## Abstract

The accumulation of protein aggregates is a hallmark of many diseases, including Alzheimer’s disease. As a major pillar of the proteostasis network, autophagy mediates the degradation of protein aggregates. The autophagy cargo receptor p62 recognizes ubiquitin on proteins and cooperates with TAX1BP1 to recruit the autophagy machinery. Paradoxically, protein aggregates are not degraded in various diseases despite p62 association. Here, we reconstituted the recognition by the autophagy receptors of physiological and pathological Tau forms. Monomeric Tau recruits p62 and TAX1BP1 via the sequential actions of the chaperone and ubiquitylation machineries. In contrast, Tau fibrils from Alzheimer’s disease brains are recognized by p62 but fail to recruit TAX1BP1. This failure is due to the masking of fibrils ubiquitin moieties by p62. Tau fibrils are resistant to deubiquitylation, and, thus, this nonproductive interaction of p62 with the fibrils is irreversible. Our results shed light on the mechanism underlying autophagy evasion by protein aggregates and their consequent accumulation in disease.

## Teaser

Alzheimer’s Tau aggregates fail to bind TAX1BP1, recruiter of the autophagy machinery responsible for aggregate clearance.

## INTRODUCTION

The maintenance of an intact proteome is essential for organismal health, as the accumulation of protein aggregates is associated with severe diseases including neurodegeneration (*1*, *2*). To counteract protein aggregation, a complex network known as the proteostasis network has evolved. Within this network, chaperones play a critical role by binding to misfolded proteins, preventing their aggregation, and promoting refolding. If refolding fails, the ubiquitin system tags the misfolded protein with ubiquitin chains, marking it for degradation by either the proteasome or selective macroautophagy (hereafter, autophagy) (*3*–*5*). The autophagic degradation of protein aggregates is termed aggrephagy (*6*). However, the interplay among the different pillars of this proteostasis network in triaging proteins toward refolding or degradation by the proteasome or selective autophagy remains unclear. Particularly enigmatic is the integration of selective autophagy into the proteostasis network despite its evident role in the degradation of protein aggregates (*4*, *5*, *7*, *8*).

For the selective degradation of ubiquitylated proteins by selective autophagy, these proteins (also referred to as cargos) are first sequestered into larger condensates by the oligomeric p62 cargo receptor (*9*–*12*). The NBR1 cargo receptor promotes this condensation reaction (*13*). NBR1 also mediates the recruitment of the TAX1BP1 cargo receptor, which in turn is the main recruiter of the upstream machinery to initiate autophagosome biogenesis at the condensates, eventually resulting in their degradation within lysosomes (*13*–*15*). Loss of TAX1BP1 leads to the accumulation of protein aggregates in the brain (*16*). Notably, biochemical reconstitutions providing mechanistic insights into the interaction of the autophagy machinery with its cargos were predominantly conducted with artificial model proteins, raising the question whether interactions between selective autophagy receptors and disease-associated protein aggregates rely on the same molecular principles (*10*, *11*, *13*, *14*).

Tau is a Janus-faced protein; in its monomeric form, it is largely unstructured and binds to microtubules (*17*, *18*). By contrast, upon aggregation, its central microtubule-binding domain refolds into a β-hairpin structure followed by oligomerization into fibrils with an amyloid core (*17*–*19*). The accumulation of these fibrils, also referred to as neurofibrillary tangles, is associated with various neurodegenerative diseases including Alzheimer’s disease (AD), the most common neurodegenerative disease (*20*). The spatial progression of Tau aggregation correlates well with the areas affected by neurodegeneration in AD brains (*21*). Monomeric Tau can be degraded by the proteasome and autophagy (*22*–*24*). Conversely, in AD brains, Tau fibrils are decorated with ubiquitin and p62 but for an unknown reason are not efficiently degraded by the autophagy machinery (*25*).

Here, we used a reconstitution approach to shed light on the molecular principles underlying Tau recognition by selective autophagy in its monomeric and fibrillar forms, the latter extracted from AD brains. We uncover how chaperones can dictate the ubiquitylation of monomeric Tau and render it a selective autophagy cargo in a ubiquitin-dependent manner. We also uncover an unexpected difference in how the autophagy receptors interact with monomeric versus fibrillar Tau. In particular, we find that TAX1BP1, the main recruiter of the upstream autophagy machinery, is not efficiently recruited to Tau fibrils. This defect results from the saturation of ubiquitin marks on fibrillar Tau by p62 and NBR1, preventing TAX1BP1 from stably interacting with the selective autophagy cargo. Moreover, we find that ubiquitylated Tau fibrils are resistant to deubiquitylation, therefore rendering the nondynamic, ubiquitylated, and p62:NBR1-coated Tau fibrils a nonproductive end point refractory to autophagic degradation.

## RESULTS

### Chaperone-dependent ubiquitylation renders Tau a selective autophagy cargo

**Fig. 1.**
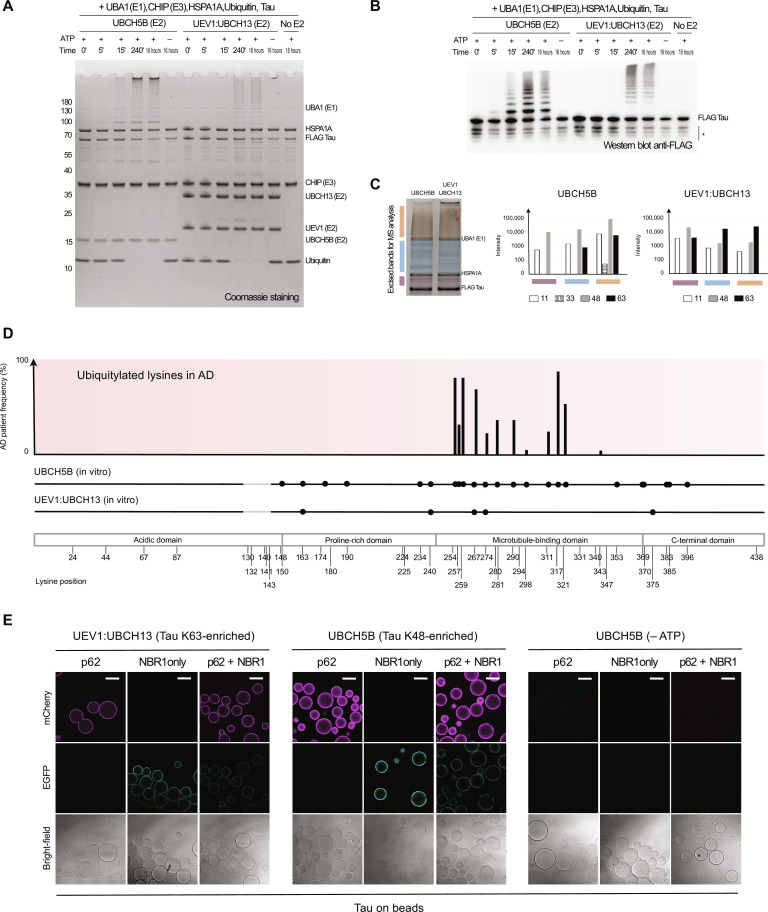
Chaperone-dependent ubiquitylation renders Tau a selective autophagy cargo. (**A**) Tau ubiquitylation by the Hsp70:CHIP machinery in the presence of different E2 enzymes (UBCH5B or UEV1:UBCH13). (**B**) Western blot anti FLAG of samples in (A). *, Tau C terminus degradation products. (**C**) Chain types assembled on Tau, found by mass spectrometry (MS) analysis and plotted for peptide intensity. Left, SDS–polyacrylamide gel electrophoresis of analyzed samples; middle and right, peptide intensity of ubiquitin linkage-specific peptides, respectively, for UBCH5B and UEV1:UBCH13. Colors indicate the bands analyzed. (**D**) Lysines targeted by the ubiquitylation machinery, found by MS analysis. Top histogram, lysines ubiquitylated in patients with AD [as described in (*29*)]; ubiquitylation map of Tau, lysines ubiquitylated in vitro are marked with black circle, respectively, for UBCH5B and UEV1:UBCH13; ubiquitylated lysines detected in both biological repeats (fig. S1C) are shown. Gray areas not covered experimentally. (**E**) Microscopy-based protein-protein interaction assay. Autophagy cargo receptors p62 and NBR1 were fused, respectively, to mCherry and enhanced green fluorescent protein (EGFP) at the N termini; ubiquitylated Tau was generated either via UEV1:UBCH13 or via UBCH5B (±ATP) and used to coat anti-FLAG beads. Scale bar, 100 μm.

To dissect the mechanisms by which the selective autophagy machinery interacts with a physiologically relevant cargo and how its activity is integrated into the general proteostasis network, we reconstituted the ubiquitylation of Tau. We mixed Tau (isoform 2N4R) with the E1 enzyme UBA1, free ubiquitin, the molecular HSP70 family chaperone HSPA1A (*26*), the E3 ligase CHIP, which was previously shown to mediate Tau ubiquitylation in cells (*27*), and two different E2s, namely UBCH5B and the dimer UEV1:UBCH13 (*28*). We monitored the assembly of ubiquitin chains over time for both E2s tested, showing faster ubiquitylation kinetics for the UBCH5B reaction within the range of minutes. In contrast, UEV1:UBCH13 ubiquitylated Tau within the range of hours (Fig. 1A). We confirmed that Tau was a substrate of ubiquitylation by blotting for its FLAG tag, located at the N terminus (Fig. 1B). The ubiquitylation kinetics for the chosen E2s were reproducible and required adenosine triphosphate (ATP), the E3 ligase CHIP, and HSPA1A (fig. S1, A and B).

Next, we determined the ubiquitin chain types attached to Tau by the different E2 enzymes. We analyzed by mass spectrometry different gel slices containing either Tau modified with one ubiquitin moiety (purple band, Fig. 1C), few units of ubiquitin (blue band), or longer ubiquitin chains (orange band). This revealed that UEV1:UBCH13 produced more K63- relative to K48-linked ubiquitin chains in comparison to UBCH5B, demonstrating how E2 selection is a powerful tool to direct ubiquitin chain–type assembly on Tau. In the following, we will refer to these two pools of ubiquitylated Tau as either K48-enriched or K63-enriched.

We went on to characterize by mass spectrometry which lysines were preferentially targeted by the ubiquitylation machinery. Tau 2N4R has 44 lysines, clustering preferentially in the microtubule-binding region (Fig. 1D, *x* axis). Our analysis revealed that both E2s targeted similar sites, located in the beginning of the microtubule-binding region and the beginning of the C-terminal domain (Fig. 1D and fig. S1C). The same first repeat of the microtubule-binding region is ubiquitylated in most patients diagnosed with AD [Fig. 1D, top, taken from (*29*)]. Thus, we were able to reconstitute the pathologically relevant ubiquitylation patterns of the microtubule-associated protein Tau in vitro. By selecting an appropriate E2 enzyme, we could also direct the Hsp70:CHIP complex to generate specific ubiquitin chain types, devising a tool to test chain-type contribution to Tau interaction with the autophagy machinery.

The sequestration of ubiquitylated cargo material by p62 and NBR1 precedes autophagic degradation (*11*, *14*, *16*, *30*). To date, reconstituting these initial steps for a physiologically relevant cargo has remained elusive. We took advantage of the generated pools of K48-enriched or K63-enriched ubiquitylated Tau to understand how p62 and NBR1 interact with this cargo protein. To this end, we generated truncations of p62 and NBR1 (fig. S2A) and turned to a microscopy-based protein-protein interaction assay, probing the interaction of fluorescently tagged p62 and NBR1 with anti-FLAG agarose beads coated with ubiquitylated Tau (fig. S1D). We detected an interaction of both full-length p62 and NBR1 with ubiquitylated Tau, irrespective of the chain type (Fig. 1E). We confirmed that differences in signal intensity were neither due to different Tau levels on coated beads nor on prey levels (fig. S2, B and C). Repeating the procedure in the absence of ATP abolished the interaction between Tau and the two autophagy receptors, pointing at Tau ubiquitylation as a necessary prerequisite for Tau interaction with the autophagy machinery. Consistent with these results and previous findings using artificial cargos, the efficient recruitment of p62 to ubiquitylated Tau required its PB1 and UBA domains, the former necessary for p62 oligomerization and the latter for binding to ubiquitin (fig. S2D, quantified in fig. S2E). On the other hand, the recruitment modalities of NBR1 were more complex. In particular, regions other than the NBR1 UBA domain promoted the interaction with ubiquitylated Tau species (fig. S2D, quantified in fig. S2E).

### Ubiquitylated Tau is a substrate for condensation by the selective autophagy receptors p62 and NBR1

**Fig. 2.**
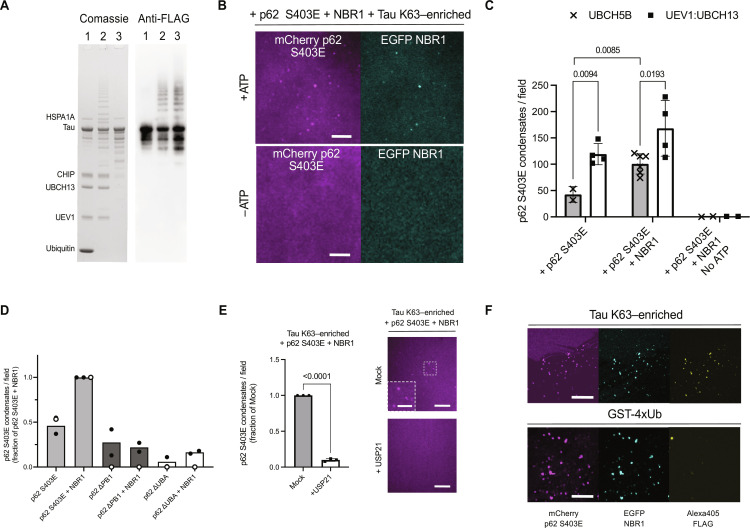
Ubiquitylated Tau is a cargo for condensation by the selective autophagy receptors p62 and NBR1. (**A**) Purification of ubiquitylated Tau (E2: UEV1:UBCH13) from the ubiquitylation machinery, verified both via Coomassie staining and Western blot against FLAG tag located at the N terminus of Tau. Lane 1: ubiquitylation reaction at 0 min; lane 2: ubiquitylation reaction at 17 hours; lane 3: eluted product after anti-FLAG resin binding. (**B**) Condensate formation in the presence of mCherry P62 S403E (2 μM), EGFP NBR1 (1 μM), and Tau ubiquitylated with UEV1:UBCH13 (±ATP), imaged via spinning disk microscopy. Scale bar, 50 μm. (**C**) Quantification of mCherry P62 S403E (2 μM) condensates plus EGFP NBR1 (1 μM) and Tau ubiquitylated with the indicated E2 (15 min for UBCH5B and 17 hours for UEV1:UBCH13). An unpaired, two-tailed Student’s *t* test was used to estimate significance; error bars, SD. (**D**) Quantification of condensates formed by ubiquitylated Tau in the presence of p62 variants (2 μM) plus EGFP NBR1 (1 μM). Tau was ubiquitylated 17 hours with the E2 UEV1:UBCH13 (white dots, GST-4xUb). (**E**) Quantification of condensates formed by ubiquitylated Tau in the presence of mCherry p62 S403E (2 μM) plus EGFP NBR1 (1 μM), ± the DUB USP21. Right, representative images for both conditions, mCherry p62 S403E imaged via spinning disk microscopy. Scale bar, 50 μm, inset, 10 μm. An unpaired, two-tailed Student’s *t* test was used to estimate significance; error bars, SD. (**F**) Colocalization of p62 S403E (2 μM), EGFP NBR1 (1 μM), and Alexa 405:FLAG with ubiquitylated Tau or GST-4xUb, assessed by confocal microscopy. Scale bar, 100 μm.

Condensation of autophagy cargo receptors with their cargos is a necessary step for subsequent clearance by selective autophagy (*13*). We therefore set out to reconstitute the condensation of p62, NBR1, and ubiquitylated Tau. We first eluted ubiquitylated Tau from agarose beads via an excess of FLAG peptide, controlling that the ubiquitylation machinery was largely removed (Fig. 2A). Upon mixing K63-enriched Tau with p62 and NBR1, we could observe condensate formation (Fig. 2B). We used a phospho-mimicking mutant of p62 with an increased affinity for ubiquitin (*31*), as confirmed by increased binding to ubiquitylated Tau on beads (fig. S2F). Repeating the ubiquitylation reaction in the absence of ATP did not yield condensates, suggesting that Tau ubiquitylation is necessary both for the interaction with, and the condensation by, the autophagy machinery. We quantified the number of condensates formed both for K63-enriched and K48-enriched ubiquitylated Tau, revealing a statistically significant higher number of condensates for the K63-enriched pool, both for p62 S403 alone and in combination with NBR1 (Fig. 2C). The preference of the autophagy receptors for K63 chains is consistent with their role in directing substrates toward the autophagy pathway (*32*, *33*).

To gain further mechanistic insights into the process of condensation, we deployed p62 truncations devoid of their individual UBA and PB1 domains, in combination with full-length NBR1 (Fig. 2D). For all conditions, we compared the Tau results with glutathione *S*-transferase (GST) fused to four M1-linked ubiquitin moieties (GST-4xUb), known to trigger robust condensation when mixed with p62 and NBR1 (Fig. 2D, white dots) (*34*). Consistent with our microscopy-based pull-down experiments, both p62 polymerization and ubiquitin-binding properties were necessary to trigger Tau K63-enriched condensation, as evidenced by the lack of condensates in the presence of p62 without either the PB1 or the UBA domain. Notably, the UBA domain of p62 is mutated in various diseases (*35*). As for GST-4xUb, NBR1 could not rescue condensation. We further determined that sub-stoichiometric amounts of ubiquitylated Tau, down to one-eighth of p62 molarity, were sufficient to trigger condensate formation (fig. S2G). We confirmed ubiquitylation as the only trigger for condensation, as treatment with the broad spectrum deubiquitylase (DUB) USP21 (*36*) completely abolished condensate formation (Fig. 2E).

Next, we confirmed that the formed condensates contained Tau by probing proteinaceous material deposited at the bottom of the reaction wells after condensation, using an antibody against the C terminus of Tau. This revealed extensive colocalization with p62 and NBR1, which was not observed neither for GST-4xUb nor for the two cargo receptors alone (Fig. 2F and fig. S2H). Together, our results demonstrate that p62 and NBR1 can sequester ubiquitylated Tau into condensates, with a preference for K63 chains. Mechanistically, both p62 polymerization and its UBA domain are necessary for condensation.

### Reconstitution of p62:NBR1 condensation with fibrillar Tau purified from postmortem AD brains

**Fig. 3.**
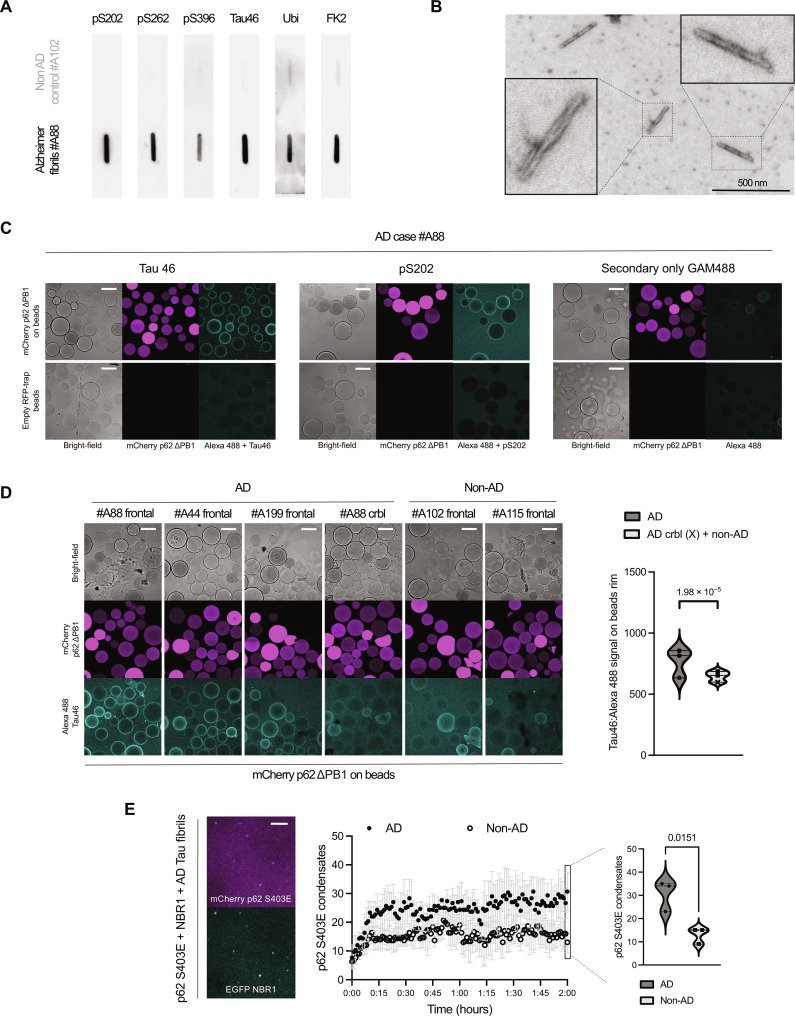
Reconstitution of p62:NBR1 condensation with fibrillar Tau purified from postmortem AD brains. (**A**) Slot blot antibody characterization of postmortem sarkosyl insoluble fraction of both one non-AD and one AD brain sections. (**B**) Electron microscopy of fibrils purified postmortem from one AD brain section. (**C**) Microscopy-based protein-protein interaction assay. Fibrillar Tau purified from an AD brain section mixed with RFP-Trap beads, either empty or coated with mCherry p62 ΔPB1. Fibrils were detected via either Alexa 488:Tau46 or Alexa 488:pS202. Scale bar, 100 μm. (**D**) Microscopy-based protein-protein interaction assay. AD or non-AD–derived sarkosyl insoluble fractions mixed with RFP-Trap beads coated with mCherry p62 ΔPB1. Fibrils were detected via Alexa 488:Tau46. Scale bar, 100 μm. Right, quantification via violin plot (dashed line, median; dotted lines, quartiles); crbl/x, cerebellum from an AD case. A nested analysis of variance (ANOVA) was used to estimate significance. (**E**) Left, representative image of mCherry P62 S403E (2 μM) and EGFP NBR1 (1 μM) condensates in the presence of an AD-derived sarkosyl insoluble fraction, visualized by spinning disk microscopy. Scale bar, 50 μm. Middle, kinetics of condensate formation of sarkosyl insoluble fractions of both non-AD and AD cases (error bars, SD). Right, quantification of condensate formation of three non-AD cases and three AD cases; violin plot (dashed line, median; dotted lines, quartiles) shows the number of condensates formed after 2 hours. An unpaired, two-tailed Student’s *t* test was used to estimate significance.

Next, we asked whether Tau aggregation associated with pathology would alter its interaction with the autophagy machinery. To this end, we purified sarkosyl insoluble Tau fibrils from frozen postmortem human AD tissue samples, following a protocol preserving ubiquitylation (*37*). As a control, we repeated all purification steps with brain tissue not containing Tau deposits. We characterized the purified extracts using a panel of antibodies against three phosphoserines known to be hyperphosphorylated in AD (*29*, *38*). As expected, extracts from a patient with AD were reactive for Tau pS202, pS262, and pS396, while a control brain extract showed no reactivity (Fig. 3A). We then probed both extracts for an antibody against the C terminus of Tau (Tau46), confirming the presence of Tau species only in the AD sample. Last, we analyzed the reactivity of both extracts against two anti-ubiquitin antibodies, either recognizing pan-ubiquitin or ubiquitin chains. Both antibodies showed stronger reactivity against the AD sample, consistent with the presence of ubiquitin moieties localized on Tau fibrils (Fig. 3A). We then repeated the extraction for another two AD cases, another two controls, and an AD case extract obtained from the cerebellum, an area known to not accumulate pathological Tau, obtaining overall consistent results in terms of antibody reactivity (fig. S3A). We further characterized fibrillar samples via transmission electron microscopy, revealing straight and paired helical filaments classically associated with AD (Fig. 3B) (*19*). With this purified Tau fibrillar material, we set out to reconstitute the initial steps of selective autophagy with these disease-associated protein aggregates.

We first tested whether postmortem Tau fibrils would directly interact with p62. To do so, we coated agarose beads with recombinant p62 ΔPB1. This mutant lacks the ability to polymerize and therefore coats the beads more homogenously than the wild-type protein. We then mixed these beads with Tau fibrils extracted from the frontal cortex of an AD case, probing fibrils with an antibody against the C terminus (Tau46) and a phosphoserine associated to disease (pS202). Both antibodies showed a specific reactivity against beads only when they were coated with p62 (Fig. 3C). Empty beads did not show any reactivity, and neither did conditions without primary antibody, strongly suggesting a specific interaction between p62 and patient-derived Tau fibrils. We expanded the assay to another two AD cases, the cerebellum of an AD case and the cortices of two non-AD cases, again probing Tau interaction with an antibody against its C terminus. Fibrils were robustly recruited to beads for all three AD cases, whereas recruitment was largely absent for non-AD cases and the AD cerebellum, devoid of Tau fibrils (Fig. 3D). Thus, we established a robust biochemical assay to probe for the interaction between Tau fibrils and p62.

Next, we set out to test whether p62 could not only interact but also condense Tau fibrils. We mixed Tau fibrils with p62 and NBR1, scoring condensate formation over time both for AD and non-AD sarkosyl insoluble extracts (Fig. 3E, middle). We observed a statistically significant increase in the number of condensates formed for all AD cases when compared to control brains (Fig. 3E, right). Of note, both p62 and NBR1 were robustly recruited to the condensates (Fig. 3E, left). Further, p62 showed no mobility for all substrates tested, as determined by fluorescent recovery after photobleaching (FRAP, fig. S3B). This is consistent with our previous work on GST-4xUb (*11*), suggesting p62 rigidity as a shared feature of physiological and pathological condensates. As for monomeric ubiquitylated Tau, we confirmed that condensates contained Tau by probing proteinaceous material deposited at the bottom of the reaction wells with an antibody against the C terminus of Tau, revealing extensive colocalization of Tau fibrils with p62 and NBR1, either with each cargo receptor alone or in combination (fig. S3C). Such colocalization was not observed for a non-AD sample. Similarly, when p62 and NBR1 were mock-treated with buffer only, or treated with GST-4xUb, such colocalization was not observed. Colocalization between the three fluorophores was evident for all AD samples and dropped for control brains and the cerebellum of a patient with AD (fig. S3D, top), indicating that p62 and NBR1 preferentially sequestered protein material containing Tau. Conversely, probing condensates with an antibody against conjugated ubiquitin gave homogeneous colocalization for all samples, indicating that ubiquitin is a major component of all sequestered species, independent of the presence of Tau fibrils (fig. S3D, bottom). Through these reconstitutions, we established that AD-derived Tau fibrils are directly recognized by the cargo receptors p62 and NBR1, representing the initial step of aggrephagy.

### p62:NBR1 condense AD Tau fibrils through ubiquitin binding, but this interaction is resistant to deubiquitylation

**Fig. 4.**
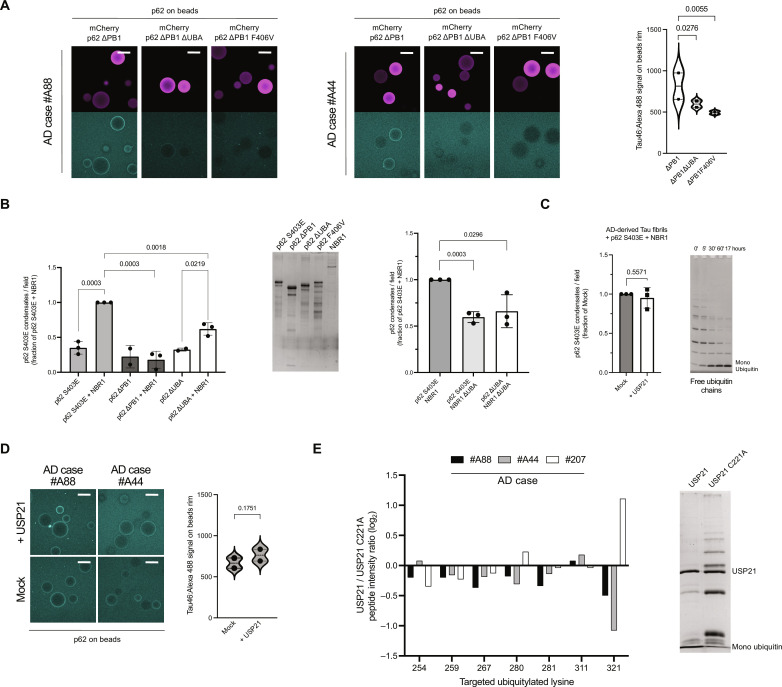
p62:NBR1 condense AD Tau fibrils through ubiquitin binding, but this interaction is resistant to deubiquitylation. (**A**) Microscopy-based protein-protein interaction assay. Fibrillar Tau from two AD brain sections (frontal) mixed with RFP-Trap beads, either coated with mCherry p62 ΔPB1, mCherry p62 ΔPB1 ΔUBA, or mCherry p62 ΔPB1 S403E. Fibrils were detected via Alexa 488:Tau46. Scale bar, 100 μm. Right, quantification of A via violin plot (dashed line, median; dotted lines, quartiles). A two-way ANOVA was used to estimate significance. (**B**) Left, fibrillar Tau condensates formation in the presence of mCherry p62 variants, ±NBR1. Middle, SDS-PAGE of input proteins. Right, as in left, in the presence of either NBR1 or NBR1 ∆UBA. An unpaired, two-tailed Student’s *t* test was used to estimate significance; error bars, SD. (**C**) Quantification of condensates formed by fibrillar Tau purified from three AD brain sections (frontal) in the presence of mCherry p62 S403E (2 μM) plus EGFP NBR1 (1 μM), ± the DUB USP21. An unpaired, two-tailed Student’s *t* test was used to estimate significance; error bars, SD. Right, SDS PAGE of USP21 activity on K48 chains. (**D**) Microscopy-based protein-protein interaction assay. Fibrillar Tau purified from two AD brain sections (frontal) mixed with RFP-Trap beads coated with mCherry p62 ΔPB1, ± the DUB USP21. Fibrils were detected via Alexa 488:Tau46. Scale bar, 100 μm. Right, quantification via violin plot (dashed line, median; dotted lines, quartiles). A nested ANOVA was used to estimate significance. (**E**) Targeted MS of seven ubiquitylated sites from three fibrillar Tau preparations purified from AD brain sections (frontal) plus either the DUB USP21 or its activity-dead mutant C221A. The *y* axis represents the log_2_ change of normalized peptide intensities (USP21 wt / C221A). Right, SDS PAGE of USP21 wt and C221A activity on K48 chains after 60 min incubation at 37°C.

We next dissected the molecular mechanism driving condensation of pathological Tau with p62 and NBR1. As our results with monomeric Tau indicated binding of p62 and NBR1 in a ubiquitin-dependent manner, we asked whether ubiquitin was also the primary cue for the recruitment of p62 and NBR1 to fibrillar Tau. Removing the UBA from p62 domain completely abolished its interaction with Tau fibrils (Fig. 4A). Using the point mutant F406V, incapable of binding ubiquitin (*39*), further confirmed the pivotal role of this posttranslational modification as the only driver of fibril recruitment to p62.

We then tested condensate formation in the presence of full-length NBR1 and p62 variants (S403E, ΔPB1, and ΔUBA). As for monomeric Tau, we observed a boost in condensation when NBR1 was added to p62 S403E (Fig. 4B, left). Oligomerization of p62 mediated by its PB1 domain was necessary for efficient condensate formation and could not be rescued by NBR1. We verified that the differences in condensation were not due to differences in cargo receptors levels (Fig. 4B, middle). As NBR1 could partially rescue condensation of p62 ∆UBA, we speculated that the NBR1 UBA domain could contribute to the rescue. To test this idea, we compared condensate formation in the presence of p62 S403E, either mixed with NBR1 or NBR1 ∆UBA. Condensation dropped significantly for the NBR1 mutant, to levels comparable to the p62 ∆UBA plus NBR1 ∆UBA combination (Fig. 4B, right). We thus concluded that the NBR1 UBA domain can contribute to condensation of fibrillar Tau.

We then tested whether condensation could be reversed by USP21 as was the case for monomeric Tau (Fig. 2E). To our surprise, Tau fibrils preincubated with USP21 still formed condensates comparable to mock samples and were therefore apparently resistant to deubiquitylation despite USP21 being active and processing ubiquitin chains (Fig. 4C). We validated this finding via a microscopy-based protein-protein interaction assay using RFP-Trap beads coated with mCherry p62 ΔPB1, observing no altered binding in the presence of USP21 (Fig. 4D, quantification on the right). This result suggested that the Tau fibrils are resistant to USP21-mediated deubiquitylation. To test this, we devised a targeted mass spectrometry approach. We set the spectrometer to scan for detectable ubiquitylated Tau peptides based on the according mass-to-charge ratio (*m*/*z*) and retention times from in vitro ubiquitylated Tau. After this method setup, we analyzed Tau fibrils from three AD brains, treated with either USP21 or its catalytically inactive version C221A. We successfully detected seven ubiquitylated lysines for all three cases, providing direct evidence of the ubiquitylation of Tau in our extracted fibrils (Fig. 4E). Furthermore, we observed no major reduction in the ubiquitylated peptide intensity ratios in the presence of active versus inactive USP21.

Together, our data strongly suggest a role of Tau fibril ubiquitylation in driving the interaction of pathological fibrils with the aggrephagy cascade. Unexpectedly, and on the contrary to monomeric Tau, the condensates containing pathological fibrils were not reversed by deubiquitylation, possibly due to fibrillar ubiquitin being inaccessible to DUBs. The catalytic domains of DUBs are much bulkier than the UBA domain of p62 and NBR1, the latter also preceded by flexible regions (fig. S4). These stark biochemical differences between Tau fibrils and monomers prompted us to dissect whether they would affect the next step of the aggrephagy cascade, namely TAX1BP1 recruitment.

### p62:NBR1 saturation of ubiquitin moieties prevents TAX1BP1 recruitment to AD Tau fibrils

**Fig. 5.**
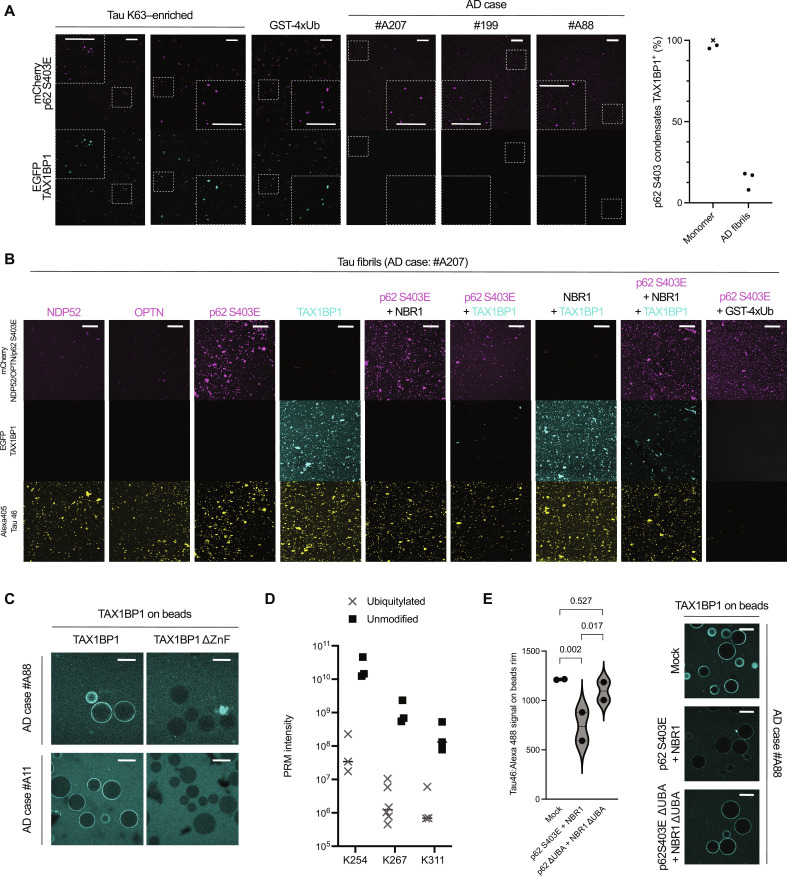
p62:NBR1 saturation of ubiquitin moieties prevents TAX1BP1 recruitment to AD Tau fibrils. (**A**) Representative images of mCherry P62 S403E (2 μM) and EGFP NBR1 (1 μM) condensates in the presence of: (i) monomeric ubiquitylated Tau K63–enriched (two independent repeats, 1 hour of condensation), (ii) GST-4xUb (2 hours of condensation), and (iii) fibrillar Tau purified from three AD brain sections (frontal, 1 hour of condensation), visualized by spinning disk microscopy. Scale bar, 50 μm. Right, colocalization score for all samples tested (*x* = GST-4xUb). (**B**) Colocalization of various autophagy cargo receptors with Tau fibrils from one AD case, assessed by confocal microscopy. Fibrils were assessed via Alexa 405:Tau46. Scale bar, 100 μm. (**C**) Microscopy-based protein-protein interaction assay. Fibrillar Tau purified from two AD brain sections (frontal, #A88; hippocampus, #A11) mixed with Glutathion Sepharose beads, either coated with GST TAX1BP1 or GST TAX1BP1 ΔZnF. Fibrils were detected via Alexa 488:Tau46. Scale bar, 100 μm. (**D**) Parallel reaction monitoring (PRM) intensity of three fibrillar Tau-specific unmodified peptides and their matching ubiquitylated versions; data extracted from Fig. 4E (plus USP21 C221A). K267 was represented by two different peptides, hence double the data points. (**E**) Microscopy-based protein-protein interaction assay. Fibrillar Tau purified from two AD brain sections (frontal, #A88; hippocampus, #A11) mixed with Glutathion Sepharose beads coated with GST TAX1BP1, plus mCherry p62 S403E (2 μM) and NBR1 (1 μM) variants. Fibrils were detected via Alexa 488:Tau46. Left, quantification via violin plot (dashed line, median; dotted lines, quartiles). A nested ANOVA was used to estimate significance. Right, representative images for each condition (brain section frontal, #A88). Scale bar, 100 μm.

TAX1BP1 was previously reported to be recruited to condensates containing p62, NBR1, and soluble ubiquitylated cargo proteins. This recruitment requires NBR1 (*13*). The recruitment of TAX1BP1 in turn was shown to be important for the subsequent recruitment of the core autophagy protein FIP200 (*13*, *15*, *16*). Of note, p62 and NBR1 form a heteropolymer with multiple p62 subunits, while TAX1BP1 is dimeric (*40*–*42*). In the presence of p62 and NBR1, TAX1BP1 was efficiently recruited to condensates triggered by either ubiquitylated K63-enriched Tau or GST-4xUb, with more than 95% of p62 condensates positive for TAX1BP1 (Fig. 5A; quantification on the right). We repeated the condensation with Tau fibrils extracted from AD brains. Unexpectedly, only a fraction of p62 condensates (<20%) contained TAX1BP1 (Fig. 5A). Imaging the bottom of the reaction wells in the presence of an antibody against the C terminus of Tau allowed us to visualize which condensates contained Tau, further confirming that only a fraction of them contained TAX1BP1 (Fig. 5B and fig. S5A). Of note, the other autophagy cargo receptors NDP52 and OPTN, classically associated with xenophagy and mitophagy (*43*, *44*), did not colocalize with the condensates (Fig. 5B and fig. S5A). Immunohistochemistry of the corresponding postmortem human tissue sections diagnosed with AD also failed to show any immunoreactivity for TAX1BP1 and only partial reactivity for NBR1 in neurofibrillary tangles. Conversely, p62 reactivity was detected, including its phosphorylated pSer349 form, which is activated in selective autophagy (fig. S6) (*45*). These in vivo results suggest a derailment of autophagy assembly on pathological Tau, consistent with our in vitro results.

We next investigated the mechanism underlying the lack of TAX1BP1 recruitment to pathological fibrils. A previous report showed the importance of its Zinc Finger (ZnF) in removing ubiquitylated aggregates in vivo (*16*). We tested the role of the ZnF domain in vitro, showing that TAX1BP1 ΔZnF is less efficiently recruited to p62:NBR1:GST-4xUb condensates compared to the wild-type protein (fig. S5B). In the case of K63-enriched Tau, TAX1BP1 ΔZnF colocalized less with p62 when compared to wild-type TAX1BP1 (fig. S5C). Thus, the ubiquitin-binding ZnF domain of TAX1BP1 is required for its robust recruitment to aggrephagy targets. Prompted by this observation, we moved to AD-derived fibrils. In a microscopy-based assay, we compared binding of fibrils to either TAX1BP1 ΔZnF or full length, observing a completely abolished interaction when the ZnF was removed (Fig. 5C). This observation, combined with the scarcity of ubiquitylation on fibrils detected by mass spectrometry (Fig. 5D), lead us to hypothesize that the p62 and NBR1 UBA domains would compete with the TAX1BP1 ZnF for the binding to ubiquitin on Tau fibrils. In contrast to soluble ubiquitylated Tau, the polymeric nature of the p62:NBR1 heteropolymer and of the cargo would render this interaction extremely stable (*34*). In particular, the p62 and NBR1 UBA domains would have a higher avidity for ubiquitin because they are present in a polymer and thus saturate all sites potentially available for the ZnF domains of the dimeric TAX1BP1, thereby excluding it from the cargo. To test this hypothesis more rigorously, we coated agarose beads with TAX1BP1 and mixed them with Tau fibrils as well as with soluble p62 and NBR1, both either full length or ΔUBA (Fig. 5E). The addition of full-length p62 or NBR1 resulted in a drastic decrease of the Tau signal on the beads, suggesting the sequestration of Tau fibrils by p62 or NBR1, thereby preventing TAX1BP1 from recruiting fibrillar Tau to the beads. When both proteins were depleted of their UBA domains, the Tau signal was restored to mock levels, confirming our hypothesis that ubiquitin on Tau fibrils has a strong preference for, and is saturated by, the UBA domains of the p62:NBR1 polymer. The effect of this saturation is the exclusion of TAX1BP1 from pathological condensates, likely due to the limited availability of ubiquitin on this cargo. Our model postulates ubiquitin scarcity on Tau fibrils. We verified that such scarcity was intrinsic to Tau fibrils and not caused by the extraction protocol. We thus repeated the procedure in the presence of the DUB inhibitors iodoacetamide and *N*-ethylmaleimide (fig. S5D). TAX1BP1 was still poorly recruited, suggesting that ubiquitin is indeed a rare posttranslational modification on Tau fibrils.

**Fig. 6.**
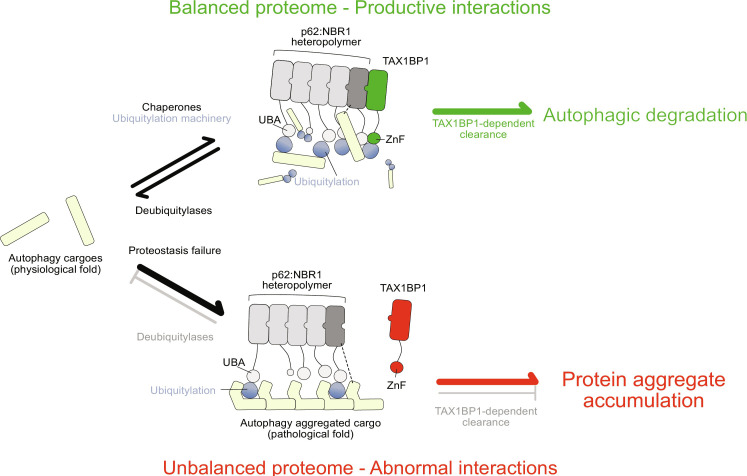
Model for the unproductive interactions between the autophagy cascade and fibrillar Tau. Monomeric Tau (represented as “autophagy cargos”) is ubiquitylated by an interplay between the chaperone and ubiquitin systems. When (mono- and/or poly-)ubiquitylated Tau accumulates, it is sequestered by the p62 and NBR1 cargo receptors (dashed lines indicate additional interaction between the cargo and NBR1). Upon recruitment of TAX1BP1, it is degraded by autophagy. In case degradation fails, condensates can be disassembled by DUBs. Pathological cargos such as fibrillar Tau are also present in assemblies containing cargo receptors. Autophagic degradation is however inefficient because TAX1BP1 is not robustly recruited due to ubiquitin moieties being saturated by p62 and NBR1. In addition, fibrils are resistant to deubiquitylation activity and further exacerbate abnormal accumulation of p62-positive pathological folds.

Together, our results provide a biochemically coherent explanation for TAX1BP1 recruitment to condensates based on its interaction with NBR1 and ubiquitin moieties on a physiological target via its ZnF. For pathological Tau fibrils, TAX1BP1 cannot access ubiquitin moieties due to their sequestration by the p62 and NBR1 UBA domains. TAX1BP1 is therefore largely excluded from pathological condensates, failing to link them to downstream autophagy (Fig. 6).

## DISCUSSION

Our study aimed to understand how selective autophagy handles physiological and pathological cargos. We demonstrated that the ubiquitylation of monomeric Tau can be directed toward the aggrephagy cascade by Tau interaction with HSP70 via the recruitment of CHIP (Fig. 1), thus establishing a cross-talk between selective autophagy and the chaperone system, two pillars of the proteostasis network (*2*). Since CHIP has been reported to promote Tau clearance in the brain (*46*), our study encourages further exploration of this cross-talk.

The interaction of CHIP with different E2 enzymes determines the preferential attachment of K48- or K63-linked ubiquitin chains to Tau. The attachment of K48 chains is markedly faster than the attachment of K63 chains, at least under the conditions tested here. K48 chains are thought to channel substrates to proteasomal degradation, while K63 chains are preferentially targeted by the autophagy machinery (*30*, *47*). Our results therefore suggest that autophagy of Tau may serve as a backup system under conditions where proteasomal degradation is impaired, matching observations in primary neurons (*48*).

Upon ubiquitylation, in particular with K63-linked chains, monomeric Tau becomes a cargo for condensation by p62 and NBR1 (Fig. 2). Condensation is strictly dependent on Tau ubiquitylation, requires oligomerization and ubiquitin binding by p62, and is promoted by NBR1. These requirements largely mirror the condensation reaction triggered by the presence of an artificial model cargo (*11*, *13*), and it can therefore be assumed that they represent a more general rule for how cargos are handled by the autophagy machinery. Of note, NBR1 binding to ubiquitylated Tau was not only dependent on its UBA domain, suggesting that specific features of the substrate may still influence selective autophagy recognition. The interaction of NBR1 with ubiquitylated Tau warrants further investigation. Condensates can be disassembled by the DUB USP21, adding reversibility to our reconstituted system, an important aspect of many physiological processes.

TAX1BP1 acts downstream of condensation and is required to initiate autophagosome biogenesis and autophagy clearance (*13*, *15*). It is also recruited to monomeric ubiquitylated Tau (Fig. 5). By reconstituting the role of its ZnF domain, we refined our current understanding of aggrephagy handover of a physiological substrate (Fig. 6, top). In particular, TAX1BP1 likely needs two molecular cues to be stably recruited to a cargo, namely its interaction with ubiquitin (via its ZnF, fig. S5B) and NBR1 (*13*). In vitro, the requirement for NBR1 can be overridden by clustering TAX1BP1 on beads or by using it at high concentrations in isolation. In vivo, the requirement of NBR1 and ubiquitin binding by TAX1BP1 may add an extra layer of regulation for the disposal of p62-positive condensates, presumably selecting the ones with a high ubiquitin load.

Our biochemical approach combining microscopy-based protein interaction assays, Tau fibrils purified from patient material and recombinant aggrephagy receptors allowed us to dissect the contribution of individual domains of the aggrephagy cascade against a highly relevant pathological cargo. While fibrillar Tau isolated from AD-affected brain regions was also accepted as cargo for p62 and NBR1-mediated condensation (Fig. 3), TAX1BP1 was recruited very inefficiently (Fig. 5). We demonstrate that this lack of recruitment is again centered on the TAX1BP1 ZnF domain, the access of which to ubiquitin moieties is impeded by their sequestration by p62 and NBR1. As ubiquitin is necessary for stable recruitment of TAX1BP1, pathological condensates are not able to attract it. The central role of the ZnF domain is strongly supported in vivo by evidence showing that its deletion results in the accumulation of ubiquitin-positive aggregates in the mouse brain (*16*). Tau fibrils may fail to hit a “ubiquitin threshold” that would render them a TAX1BP1 substrate, therefore escaping from autophagic clearance. These aberrant, nonproductive interactions between p62, NBR1, and Tau fibrils result in the accumulation of p62-positive aggregates and are supported by neuropathological observations in human AD brain immunohistochemistry (*25*). Notably, p62 is the most abundant cargo receptor compared to NBR1 and TAX1BP1 (*49*). Other proteinaceous inclusions are also p62 positive but are not degraded (*50*), suggesting that the nonproductive interaction of cargo receptors with protein aggregates may be a widespread phenomenon. Furthermore, fibrillar polyQ aggregates have been reported to be nonpreferential cargos for autophagy (*51*). A similar nonproductive sequestration with protein aggregates has also been suggested for other components of the proteostasis network (*52*–*54*).

A second important difference between physiological and pathological condensates, other than their recruitment of TAX1BP1, was their different reactivity with regard to deubiquitylation (Fig. 4). Our targeted mass spectrometry revealed that Tau fibrils are resistant to the DUB USP21. This result offers a biochemical explanation for the presence of ubiquitin on Tau fibrils from AD brains (*25*), albeit at low levels. Resistance to deubiquitylation is likely upstream of condensate formation and contributes to a vicious cycle where nondynamic Tau fibrils sequester p62. In turn, these fibrils are not cleared by TAX1BP1-dependent autophagy, resulting in their accumulation (Fig. 6, bottom). Consistently, recent work in cell lines showed that ubiquitylated aggregated Tau is resistant to autophagic clearance (*55*).

In summary, our study provides the reconstitution of the selective autophagy machinery in combination with a physiological and a pathological cargo. Further studies should test our model in cellulo and in vivo. Nonetheless, we offer a mechanistic explanation for why pathological Tau is no longer cleared effectively by selective autophagy. Moreover, our work may also guide future approaches designed to artificially induce the degradation of proteins aggregates—including Tau fibrils—in cells, for instance by tethering TAX1BP1 rather than p62 to pathological cargos.

## MATERIALS AND METHODS

### Cloning and protein production

FLAG-Tau, UBA1, UBCH5B, HSPA1A, CHIP, ubiquitin, mCherry p62, mCherry p62 S403E, mCherry p62 ΔPB1, mCherry p62 ΔUBA, mCherry p62 ΔPB1 ΔUBA, mCherry p62 ΔPB1 F406V, mCherry p62 F406V, mCherry OPTN, and mCherry NDP52(P389A) were cloned into a pETDuet plasmid backbone (for a complete list of plasmids, see Table 1). UEV1 was cloned into a p27a plasmid backbone with an His tag at its N terminus. UBCH13 was cloned into a pET-SUMO plasmid backbone. 4xUb was cloned in a pGEX-4 T1 plasmid backbone. The obtained plasmids were transformed in *Escherichia coli* Rosetta cells by heat shock for 45 s at 42°C and expressed in LB medium containing the proper antibiotics. Cultures were then scaled up to the range of liters, grown until optical density at 600 nm reached 0.6 to 1, and induced with 0.15 mM isopropylthiogalactoside (IPTG). Cells were then grown at 25°C for 5 hours (p62 constructs) or 18°C for 17 hours (all other constructs). After appropriate growth time, cells were harvested and pellets stored at −80°C in lysis buffer (Table 2).

**Table 1.** Constructs used in this study.

Protein	Construct code
Flag-Tau	SMC1368
UBA1	SMC915
UEV1	SMC883
SUMO-UBCH13	SMC885
UBCH5B	SMC1365
HSPA1A	SMC1473
CHIP	SMC1375
Ubiquitin	SMC907
mCherry p62	SMC391
mCherry p62 S403E	SMC709
mCherry p62 ΔPB1	SMC546
mCherry p62 ΔUBA	SMC590
mCherry p62 ΔPB1 ΔUBA	SMC596
mCherry p62 ΔPB1 F406V	SMC2062
EGFP-NBR1 (from insect cells)	SMC914
EGFP-NBR1 [from human embryonic kidney (HEK) cells]	SMC1810
NBR1 (from HEK cells)	SMC1811
EGFP-C-NBR1 K499-Y966 (from insect cells)	SMC1238
EGFP-NBR1-ΔUBA (from insect cells)	SMC985
NBR1-ΔUBA (from HEK cells)	SMC2135
EGFP TAX1BP1	SMC1435
EGFP TAX1BP1 ΔZnF	SMC2144
GST TAX1BP1	SMC1434
GST TAX1BP1 ΔZnF	SMC2143
mCherry OPTN	SMC397
mCherry NDP52	SMC1148
GST-4xUb	SMC538
USP21	SMC947
USP21 C221A	SMC1271

**Table 2.** Purification buffers for NBR1 variants.

Reagent (stock)	Lysis buffer	His trap, A	His trap, B	SEC buffer
Hepes (pH 7.5) (1 M)	50 mM	50 mM	50 mM	25 mM
NaCl (5 M)	150 mM	150 mM	150 mM	150 mM
MgCl_2_ (1 M)	2 mM	-	-	-
CHAPS	0.25%	0.25%	-	-
Protease inhibitor (Roche, EDTA free)	1 tablet/50 ml	-	-	-
PefaBlock (100 mM)	1 mM	-	-	-
Benzonase (MilliporeSIGMA)	1 μl/50 ml	-	-	-
DNAse (Sigma-Aldrich)	A 0.2 g/50 ml	-	-	-
RNAse	20 μl/50 ml	-	-	-
Protease inhibitor cocktail (Sigma-Aldrich)	150 μl/50 ml	-	-	-
Imidazole (3 M)	10 mM	10 mM	300 mM	-
β-Mercaptoethanol (14.3 M Sigma-Aldrich)	2 mM	2 mM	2 mM	-
DTT (1 M)	-	-	-	1 mM

Enhanced green fluorescent protein (EGFP)–NBR1, EGFP-NBR1 K499-Y966, and EGFP NBR1 ΔUBA were cloned into a FastBac plasmid backbone, all constructs with an His-Strep-TEV sequence at the N terminus. The obtained plasmids were transformed into electro-competent *E. coli* DH10BacY cells. One microliter of plasmid with a concentration of 25 ng/μl was electroporated with a Bio-Rad MicroPulser with setting for bacteria (two times 5.8 ms at 1.8 kV). After recovery at 37°C for 5 hours in LB medium, cells were plated on plates with kanamycin (50 μg/ml), gentamicin (7 μg/ml), tetracycline (10 μg/ml), X-galactosidase (200 μg/ml), and 165 μM IPTG. White colonies were selected, and the recombinants were checked by polymerase chain reaction with insert-specific primers M13FW (SMP1770) and M13RV (SMP1771). The verified bacmids were then prepped and used for transfection of the Sf9 insect cells for baculovirus production. Insect cells were cultivated in insect cell culture medium (ESF 921, Expression System LLC) supplemented with penicillin-streptomycin at 27°C in humidified 5% CO_2_ atmosphere. About 1 million insect cells were plated in one well of a six-well plate with 2.5 ml of regular media with antibiotics. Bacmid DNA (2.5 μg) was transfected with the help of 5 μl of FuGENE HD (Promega, no. E2312), and the expression of protein of interest was directly observed by the expression of yellow fluorescent protein. The V0 virus was harvested by collecting the supernatant in the well after 7 to 8 days and used to transfect a 30-ml insect cell culture. V1 virus was harvested when the viability of cells dropped from almost 100 to 90%, stored at 4°C for up to 6 months after filtration. The V1 virus was then used to infect large volumes of cell culture for protein production, with cells harvested when viability dropped to 90%. Pellets were stored at −80°C. These NBR1 variants were used in Figs. 1E and 2 (B, C, and F) and figs. S2 (C, D, E, and H).

NBR1, EGFP NBR1, and EGFP NBR1 ΔUBA were cloned in a pCAGGSsH-C vector and expressed in human embryonic kidney 293. These NBR1 variants were used in Figs. 2D, 4, and 5 and figs. S2G, S3, and S5.

### Protein purification

The buffers used in purification of NBR1 variants are shown in Table 2. Before purification, the pellets were solubilized in 50 ml of lysis buffer. The resuspended pellet was driven through a precooled glass Douncer—10 times with pestle A and 10 times with pestle B—and then sonicated (Bandelin Sonoplus) once for 45 s with 40% power to lyse the cells. The cell lysate was spun down 40 min, 186,000*g* at 4°C. The supernatant was 0.22-μm filtered (Cytiva, no. 6878-2502) and loaded on a 5-ml HisTrap column (Cytiva, no. 28907548) on an ÄKTA Fast Protein Liquid Chromatography system. The column was preequilibrated in buffer A. The imidazole elution of the HisTrap purification consisted of six buffer steps with 50, 75, 100, 150, 200, and 300 mM of imidazole, 5 column volume each. The peak fractions were checked on SDS–polyacrylamide gel electrophoresis (SDS-PAGE) and collected. Protein-containing peak fractions were pooled and, depending on the batch, either left overnight at 4°C or cleaved with His-TEV protease overnight rolling at 4°C. The gel filtration column S6 10-300 (not increase, Cytiva, no. 17517201) was preequilibrated with size exclusion chromatography (SEC) buffer. The cut product was concentrated to 0.5 to 1 ml with a 100-k Amicon Ultra 15-ml centrifugal filter units (Merck, no. UFC910024) and loaded on an SEC with 1-ml loop and further separated by size through isocratic elution. The purified protein was concentrated with 100-k Amicon Ultra 4-ml centrifugal filter units. Final concentration was determined by Nanodrop A280, and the protein was aliquoted, frozen with liquid nitrogen, and stored at −80°C.

Purification of FLAG-Tau, UBA1, UBCH5B, HSPA1A, CHIP, ubiquitin, UEV1, UBCH13 mCherry p62, mCherry p62 S403E, mCherry p62 ΔPB1, mCherry p62 ΔUBA, mCherry p62 ΔPB1 ΔUBA, mCherry p62 ΔPB1 F406V, mCherry p62 F406V, mCherry OPTN, and mCherry NDP52(P389A) were purified with similar procedures. No Douncer was used, and, instead, lysates were sonicated three times 30 s each, 65% power, and five cycles. His Trap and SEC followed a procedure similar to the one described for NBR1 proteins, with the following exceptions: (i) Lysis buffer contained 25 mM Hepes (pH 7.5), 150 mM NaCl, 25 mM imidazole, 1 tablet of EDTA-free cocktail inhibitor per 50 ml of buffer, and 2 mM β-Mercaptoethanol; (ii) UEV1 and UBCH13 were coincubated 17 hours at 4°C in a 1:1 stoichiometry before SEC; (iii) all p62 buffers contained 500 mM NaCl instead of 150 mM. (iv). For FLAG-Tau, an extra step of cation exchange chromatography was added between the HisTrap and the SEC runs. Equilibration buffer contained 25 mM Hepes (pH 7.5), 45 mM NaCl, and 1 mM dithiothreitol (DTT), while elution buffer contained 25 mM Hepes (pH 7.5), 500 mM salt, and 1 mM DTT. (v) For CHIP, an extra step of anion exchange chromatography was added between the HisTrap and the SEC runs. Equilibration buffer contained 25 mM Hepes (pH 7.5), 45 mM NaCl, and 1 mM DTT, while elution buffer contained 25 mM Hepes (pH 7.5), 500 mM salt, and 1 mM DTT; (vi) all SEC steps used an S200 column 10 300 (Cytiva, no. 28990944). The concentration of the purified proteins was conducted using Amicon ultracentrifugal filtration units of the recommended cutoff pore size.

GST-4xUb, EGFP TAX1BP1, EGFP TAX1BP1 ΔZnF, GST TAX1BP1, and GST TAX1BP1 ΔZnF were produced and purified as previously described (*11*, *13*). USP21 and USP21 C221A were produced and purified according to a published protocol (*36*).

### Ubiquitylation assay

Ubiquitylation buffer (10×) consisted of 250 mM tris-HCl (pH 7.5), 1200 mM NaCl, and 10 mM MgCl_2_. DTT was added fresh to reach a working concentration of 3 mM DTT. The proteins were thawed on hand spun down 10 min at 4°C max speed (14,500*g* bench centrifuge). The reaction mix was prepared on ice following Table 3 except for ATP. The reaction was started by adding ATP and gently mixing. A sample for SDS-PAGE was taken at time point 0 min, and then the reaction mix was incubated at 30°C. After defined time intervals, samples were further processed either for SDS-PAGE analysis or flash frozen in liquid nitrogen.

**Table 3.** Ubiquitylation reaction mix components.

Reagent	Final concentration
Water	to 600 μl
Ubiquitylation buffer 10×	25 mM
E1 UBA1 (4.07 μM)	50 nM
Ubiquitin (822 μM)	100 μM
E2 UEV1:UBCH13 (350 μM)	5 μM
E3 CHIP (160 μM)	2.5 μM
HSPA1A (132 μM)	1 μM
FLAG-Tau (68.5 μM)	10 μM
ATP (pH 6) 100 mM	5 mM

Ubiquitin K48 chains were purified as previously described (*11*).

### Mass spectrometry—Sample processing of monomeric ubiquitylated Tau

Coomassie stained gel bands were cut and destained with a mixture of acetonitrile (ACN) and 50 mM ammonium bicarbonate. Disulfide bridges were reduced using DTT, and free sulfhydryl groups were subsequently alkylated by chloracetamide. The digestion with trypsin was carried out overnight at 37°C and was stopped by adding 10% formic acid to an end concentration of approximately 5%. Peptides were extracted from the gel with 5% formic acid by repeated sonication.

### Mass spectrometry—Sample processing of postmortem Tau fibrils

Fibril preperations were processed with the iST kit (PreOmics) with adaptations of the protocol due to the limited sample amount. For 1 μg of fibrils in 5 μl solution equal volumes (5 μl) of 2× LYSE buffer of the solutions RESUSPEND and DIGEST were used. Samples were digested for 3 hours at 37°C. Thirty microliters of the STOP solution was added to stop the reaction. Wash and elution steps were performed according to the manufacturer’s protocol.

### Mass spectrometry—Nano liquid chromatography tandem mass spectrometry analysis of monomeric ubiquitylated Tau

Peptides were separated on an Ultimate 3000 RSLC nano–high-performance liquid chromatography (nano-HPLC) system using a precolumn for sample loading (Acclaim PepMap C18, 2 cm by 0.1 mm, 5 μm) and a C18 analytical column (Acclaim PepMap C18, 50 cm by 0.75 mm, 2 μm, all HPLC parts, Thermo Fisher Scientific), applying a linear gradient from 2 to 35% solvent B (80% ACN, 0.1% formic acid; solvent A 0.1% formic acid) at a flow rate of 230 nl/min over 60 min. Eluting peptides were analyzed on an Orbitrap Exploris 480 mass spectrometer coupled to the HPLC via the Nanospray Flex ion-source (all Thermo Fisher Scientific) equipped with coated emitter tips (MS Wil).

The mass spectrometer was operated in data-dependent acquisition mode. Survey scans were obtained in a mass range of 375 to 1400 *m*/*z* with lock mass off, at a resolution of 120,000 at 200 *m*/*z* and a normalized Automated Gain Control (AGC) target of 300%. Over a cycle of 2 s, the most intense ions were selected with an isolation width of 1.2 *m*/*z*, for max. 100 ms at a normalized AGC target of 200% and then fragmented in the Higher-energy Collisional Dissociation (HCD) cell at 28% normalized collision energy. Spectra were recorded at a resolution of 15,000. Peptides with a charge of +1 or > +6 were excluded from fragmentation, the MIPS mode was set to “Peptide,” the exclude isotope feature was enabled, and selected precursors were dynamically excluded from repeated sampling for 20 s.

### Mass spectrometry—Nano liquid chromatography tandem mass spectrometry analysis of postmortem Tau fibrils

Fibril digests were separated on an Ultimate 3000 RSLC nano-HPLC system (the same setup as for the in-gel digests) applying a linear ACN gradient over 60 min and analyzed on an Orbitrap Exploris 480 without Field Asymmetric waveform Ion Mobility Spectrometry (FAIMS) coupled via a NanoFlex ion source to the LC (all Thermo Fisher Scientific). Survey scans were obtained in a mass range of 375 to 1500 *m*/*z* with lock mass off, at a resolution of 60,000 at 200 *m*/*z* and a normalized AGC target of 300%. The parallel reaction monitoring (PRM) parameters—precursor *m*/*z* and retention time—were built on the basis of the in vitro–ubiquitylated Tau samples. Precursor of ubiquitylated peptides and nonmodified peptides were isolated in a 0.7-*m*/*z* window and fragmented with 30% HCD collision energy. Orbitrap resolution was set to 60,000 and the normalized AGC target to 200%. Maximal injection time for modified peptides was set to 200 ms and 100 ms for unmodified peptides. The retention time windows for the PRMs were set the way that cycle time did not exceed 2 s. To avoid sample carryover, blanks were analyzed between every PRM run.

### Mass spectrometry—Data analysis of monomeric ubiquitylated Tau

Raw data were processed using the MaxQuant software package [version 1.5.2.8, (*56*)] and searched against the protein sequences of the ubiquitinylation assay in the *E. coli* proteome background (release 2019_01 UP000000625, www.uniprot.org) as well as a database of most common contaminants. The search was performed with standard identification settings: full trypsin specificity allowing a maximum of three missed cleavages. Carbamidomethylation of cysteine residues was set as fixed and oxidation of methionine, acetylation of protein N termini, and Gly-Gly on lysines as variable modifications. All other settings were left at default. Results were filtered at a false discovery rate of 1% at protein and peptide spectrum match level. For quantitative comparisons of the ubiquitin sites, site intensities were normalized by the intensity of the respective protein in the corresponding gel section.

### Mass spectrometry—Data analysis of PRMs from postmortem Tau fibrils

PRM data were analyzed and validated in Skyline-daily (version 23.09.239, MacCoss Lab Software, University of Washington, Seattle, WA) (*57*). All peptide transitions were manually evaluated for elution times, mass accuracy, and interfering signals. Peak boundaries were manually inspected and reassigned to ensure accurate integration. Seven to 10 transitions per peptide were included for quantification across sample pairs (non-DUB/DUB). PRM intensities of the modified peptides were normalized within the sample pairs based on the sum of the PRM intensities of five unmodified Tau peptides.

### Microscopy-based pull down

Microscopy-based pull down were performed following an established procedure (*58*) exemplified in fig. S1D. Bait-pray incubation was performed for 17 hours at 4°C in 384-well plates (plates, Greiner Bio-One, no. 781892; beads, RFP-Trap beads, Chromotek no. rta-20; Sepharose beads, Cytiva no. 17075605). Samples containing fibrils were first stained with Tau46 (CST, no. 4019S) 1.5 hours at room temperature (RT) and then with a secondary goat anti-mouse antibody Alexa 488–conjugated, 1 hour at RT.

Images were analyzed with an artificial intelligence–designed ad hoc for this purpose. Signal intensities in images of beads were quantified by drawing line profiles across beads drawn in Fiji (*59*) and recording the difference between the minimum and maximum gray values along the lines. To facilitate drawing a large number of lines, a Fiji plugin was designed. The plugin uses cellpose (*60*) to detect beads in an image by artificial intelligence. Processing is done in two parts, with the first operating in batch mode. Multichannel input images are split into individual TIFF images and passed to cellpose (running in a Python environment). The label images produced by cellpose are reassembled into multichannel images. Circular regions of interest (ROIs) are fitted to the segmented particles, and a predefined number of line profiles are automatically drawn starting at the ROI center and extending beyond the border of the circular ROI. A combined ROI containing all detected beads is used to exclude line profiles protruding into an adjacent bead. The second part of the plugin, operating on a single multichannel image, allows users to inspect the line profiles, to interactively add undetected beads, to modify profiles, or to exclude individual ones from the final analysis.

### M2 anti-FLAG bead purification of FLAG-Tau (ubiquitylated and monomeric)

The volume of beads slur (anti-FLAG M2 affinity Gel, Thermo Fisher Scientific, no. A2220) necessary to bind Tau was calculated according to the manufacturer’s information and then doubled to ensure that all FLAG-Tau was collected to minimize protein loss. The ubiquitylation reaction (see above, typically 600 μl) was spun down 10 min at 4°C, 14,500*g*. In the meanwhile, beads were cleaned four times with 0.8 ml of SEC buffer, vortexed for 5 s, spun down at 7000*g* for 60 s each time. Supernatant was carefully removed. Next, Tau ubiquitylation reaction was added to the equilibrated beads and incubated for 60 min on roller at 4°C. Beads were then spun down at 7000*g* for 60 s, and the supernatant was carefully removed. Beads were then washed 30 min in a cold room with SEC buffer + 5 mM ATP + 5 mM MgCl2; after that, the supernatant was carefully removed. Beads were washed once with 1 ml of SEC buffer with 5 mM ATP, vortexed for 5 s, and spun down at 7000*g* for 60 s; then, the supernatant was carefully removed. Beads were washed another two times with SEC buffer. Elution was achieved by adding FLAG-peptide (Thermo Fisher Scientific, no. F3290), stoichiometry 2:1 (FLAG-peptide:Tau), 1 hour at RT. The mix was then centrifuged for 1 min at 7000*g*. The supernatant was carefully transferred to a 35-kD dialysis membrane (Spectra standard RC tubing) without beads and dialyzed overnight at 4°C. The dialyzed protein was transferred to a 3-kDa cutoff Amicon concentrator preequilibrated with SEC buffer and centrifuged until the volume was around 100 μl. A sample for SDS-PAGE was taken for the final product, and the purified protein was aliquoted and frozen in liquid nitrogen.

### Microscopy of condensate formation

Proteins were thawed from −80°C and spun down for 10 min at 4°C of 16,000*g*. Proteins were diluted to the desired concentrations in the reaction mix, and the salt concentration was adjusted with SEC buffer to 150 mM. Buffers and diluted proteins were pipetted and mixed directly in a 384-well plate, making sure that the liquid was distributed homogenously at the bottom of the well, with a typical final volume between 10 and 20 μl.

The reaction was observed and recorded with a Visitron spinning disk microscopy after incubation for the intended length of the experiment. The camera used was EM-CCD with 20× objective, and laser intensity was set to 25% for mCherry and 25% for GFP, exposure 100, and gain 100 to 150. The stages were selected in the middle of the wells, and three stacks at 30-μm distance were recorded. Condensate formation was quantified using a previously published ImageJ macro (*11*). Figure 5A and figs. S2G and S5 (C and D) were background-corrected with Fiji Rolling Ball feature, with a pixel radius respectively of 2, 1 and 1 pixel.

To visualize the FRAP of Tau-induced p62 condensates in vitro, reactions were set up as previously described in Results. Condensates at the bottom of the 384-well plate were bleached with a 488-nm or 561-nm laser and imaged using a Visitron Spinning disk microscope equipped with a Plan-Apochromat 63×/1.4 oil immersion lens. After bleaching, the recovery of the ROI was observed for 30 s, acquiring 1 image/s.

### Immunofluorescence staining of condensates

After condensates formed, primary antibody mouse anti-Flag (6B2 clone from Ogris group, Max Perutz Labs) or Tau46 (CST, no. 4019S) was added to the well and incubated overnight at 4°C. Secondary antibody goat anti mouse IgG H&L Alexa Fluor 405 was used at a concentration of 1:500. Colocalization of the three fluorophores (mCherry of p62, EGFP of NBR1/TAX1BP1, and Alexa 405) was observed on the bottom of the wells and recorded by ZEISS LSM 700 confocal laser scanning microscope.

### Tau fibrils extraction from postmortem human brains

Tau fibrils were extracted from fresh-frozen human postmortem brains (frontal cortex) stored at −80°C with neuropathologically confirmed AD-related changes [case A88: female, 61 years, “ABC-Score” (*61*): A2B3C2, postmortem delay (PMD) 36 hours; case A199: male, 62 years, A3B3C3, PMD 48 hours; case A44: female, 88 years, A3B3C3, PMD 17 hours; case A61: female, 79 years, PMD 6 hours; case A229: female, 84 years, PMD n.a.] or controls without Tau pathology (case A102: male, 49 years, pulmonary fibrosis, PMD 16 hours; case A115: female, 35 years, Ewing sarcoma and sepsis, PMD 12 hours; case 111: male, 66 years, cardiac arrest, PMD 7 hours), following an established procedure that would preserve ubiquitin moieties on Tau fibrils (*37*). Cases no. A76 (female, 92 years, PMD 48 hours) and no. A161 (female, 75 years, PMD 15.75 hours) were extracted in the presence of 1 mM iodoacetamide and 5 mM *N*-ethylmaleimide. The extracted material was then characterized by transmission electron microscopy and slot blot analysis. Work with human samples was approved by the Ethik Kommission Medizinische Universität Wien (EK Nr: 1454/2018).

### Transmission electron microscopy

Sample (3.5 μl) was absorbed on grids for 30 s and subsequently stained shortly with 2% uranyl acetate, followed by a second staining of 40 s. Excess of solution was blotted away, and grids were left drying for at least 30 min. Grids were imaged using an FEI Morgagni operated at 100 kV.

### Slot blot

A piece of nitrocellulose membrane (no. 10600009, Sigma-Aldrich) was washed with tris-buffered saline (TBS) and mounted on slot blot manifold (PR648, Sigma-Aldrich). Fifty microliters of the sample was loaded per well, containing between 200 and 400 ng of protein material (measured via Bradford). After vacuum was applied, membrane was washed with TBS thrice and blocked in 3% milk TBS for 45 min. The following antibodies were used overnight at 4°C:

pS202 (Cell Signaling Technology, rabbit, 1:1000, no. 39357), pS262 (Thermo Fisher Scientific, rabbit, 1:250, no. 44-750G), pS396 (Thermo Fisher Scientific, rabbit, 1:1000, no. 44-752G), Tau46, amino acids 404 to 441 (CST, mouse, 1:1000, no. 4019), FK2 (Enzo Life Sciences, mouse, 1:1000, no. BML-PW8810-0500), and Ubiquitin (made in-house, Bachmair Laboratory, Max Perutz Labs, rabbit, 1:1000).

After 16 hours, the membrane was washed twice with TBS and probed with a secondary antibody conjugated to horseradish perodixidase (Jackson ImmunoResearch, no. 115-035-003). Blots were imaged via chemiluminescence with a ChemiDoc.

### Immunohistochemistry of postmortem human brains with antibodies against autophagy cargo receptors

Five-micrometer-thick formalin-fixed, paraffin-embedded tissue sections from the hippocampus of a patient with advanced AD neuropathological changes were first stained with hematoxylin and eosin followed by immunohistochemistry applying a panel of different primary antibodies after antigen retrieval (Table 4). Immunoreaction was visualized by the DAKO Envision System kit (DAKO, Glostrup, Denmark) using a Dako-Autostainer 48 Link platform. Diaminobenzidine was used as chromogen.

**Table 4.** Antibodies and pretreatments methods used for immunohistochemical staining on 5-μm-thick paraffin sections.

Antibody name	Clone	Species	Immunogen	Company	Pretreatment	Dilution
Anti-p62	Clone 3/p62 lck ligand	Mouse	Human p62 lck ligand amino acid sequence 257-437	BD Transduction Laboratories, Franklin Lakes, NJ, USA	Boiling in citrate buffer at pH6 for 20 min	1:500
Anti-phosphorylated Tau	Clone AT8, pS202/pT205	Mouse	Partially purified human PHF-Tau	Thermo Fisher Scientific, Rockford, IL, USA	None	1:200
Anti–alpha-Synuclein	Clone 5G4	Mouse	Amino acid sequence 44-57	Roboscreen, Leipzig, Germany	Boiling in citrate buffer at pH6 for 20 min, 1 min in concentrated formic acid	1:4000
Anti-ubiquitin	Clone Ubi-1	Mouse	Purified ubiquitin from bovine red blood cells covalently coupled to KLH using glutaraldehyde	Millipore, Temecula, CA, USA	Boiling in citrate buffer at pH6 for 20 min	1:50,000
Anti-p62 S349	-	Rabbit	Synthetic phosphopeptide corresponding to residues surrounding Ser^349^ of human SQSTM1/p62 protein	Cell Signaling Technology, Leiden, the Netherlands	Boiling in citrate buffer at pH6 for 20 min	1:100
Anti-TAX1BP1	D1D5	Rabbit	Synthetic peptide corresponding to residues near the carboxy terminus of human TAX1BP1 protein	Cell Signaling Technology, Leiden, the Netherlands	Boiling in citrate buffer at pH6 for 20 min	1:100
Anti-NBR1	6B11	Rabbit	Synthetic peptide of human NBR1	Abnova	Boiling in citrate buffer at pH6 for 20 min	1:50
Anti-p62 S403	D8D67	Rabbit	Synthetic phosphopeptide corresponding to residues surrounding Ser^304^ of human SQSTM1/p62 protein	Cell Signaling Technology, Leiden, the Netherlands	Boiling in citrate buffer at pH6 for 20 min	1:100

### Statistical analysis

The following description applies to all the microscopy-based pull downs quantified in this study. For each bead, the mean fluorescence and SD for all points of measurement were recorded. Beads that had an SD of point measurements equal or greater to half the mean value were excluded. Plots show the mean values for each bead corresponding to their respective experiment. Significance values were calculated for average bead fluorescence intensity through a linear mixed model in R (version >4.2) using the nlme and emmeans packages (*62*, *63*), implementing the tidyverse and rstatix packages (*64*, *65*), categorizing both experiment replicate and well number as random factors, as described in statistical guidelines (*66*). Remaining statistics was performed using GraphPad Prism 10.
