# Supplementary material for: Tau fibrils evade autophagy by excessive p62 coating and TAX1BP1 exclusion
Source: Sci Adv. 2024 Jun 12;10(24):eadm8449. doi: 10.1126/sciadv.adm8449 (PMC11168460; doi:10.1126/sciadv.adm8449)
Supplement: Supplementary file 1 — Figs. S1 to S6 [file sciadv.adm8449_sm.pdf]

Science Advances
AAAS

Supplementary Materials for

Tau fibrils evade autophagy by excessive p62 coating and TAX1BP1 exclusion

Luca Ferrari *et al.*

Corresponding author: Luca Ferrari, luca.ferrari@univie.ac.at; Sascha Martens, sascha.martens@univie.ac.at

Sci. Adv. **10**, eadm8449 (2024)
DOI: 10.1126/sciadv.adm8449

This PDF file includes:

Figs. S1 to S6

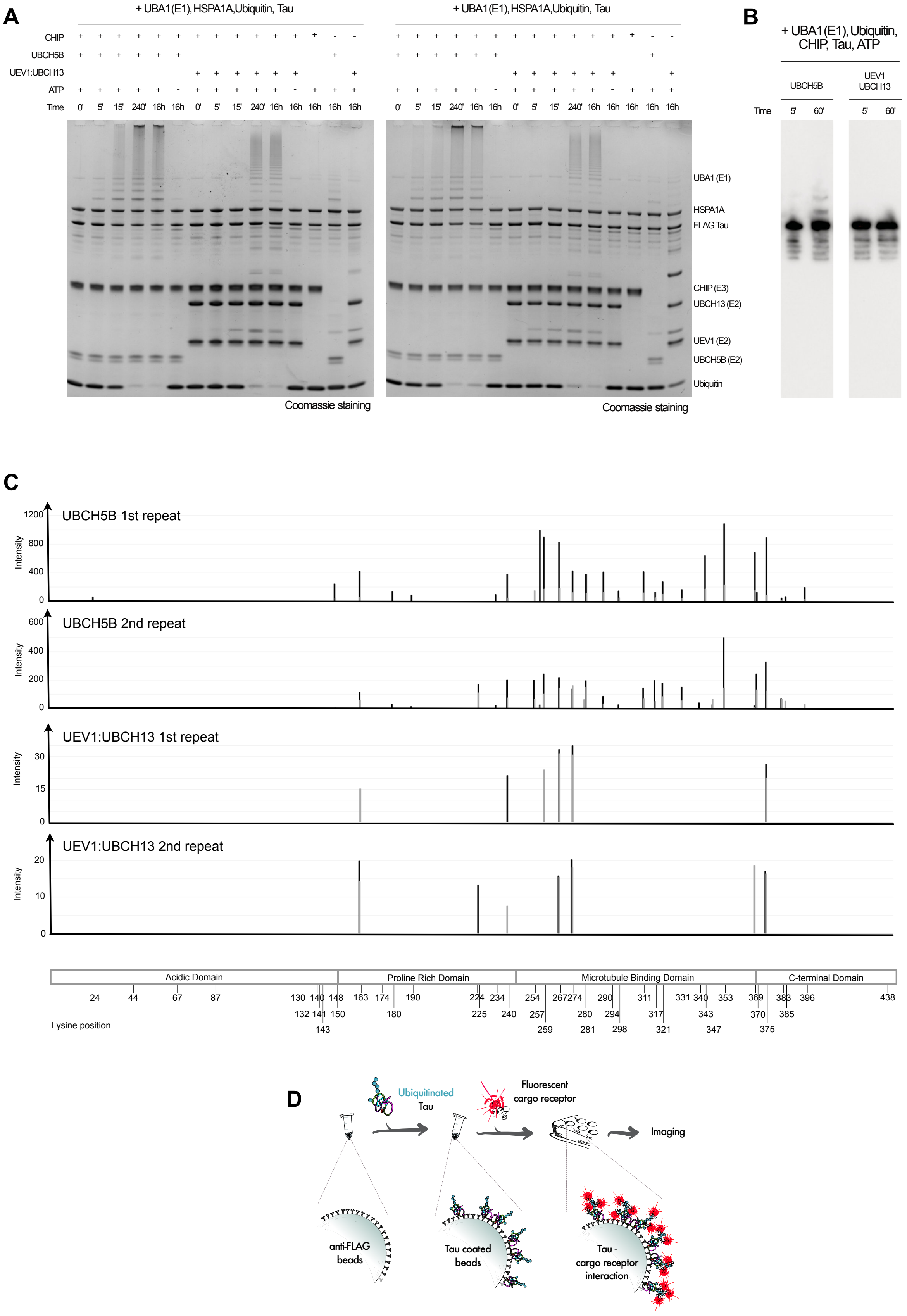

A
+ UBA1 (E1), HSPA1A,Ubiquitin, Tau
+ UBA1 (E1), HSPA1A,Ubiquitin, Tau
CHIP
UBCH5B
UEV1:UBCH13
ATP
Time
0' 5' 15' 240' 16h 16h 0' 5' 15' 240' 16h 16h 16h 16h 16h
UBA1 (E1)
HSPA1A
FLAG Tau
CHIP (E3)
UBCH13 (E2)
UEV1 (E2)
UBCH5B (E2)
Ubiquitin
Coomassie staining
Coomassie staining
B
+ UBA1 (E1), Ubiquitin, CHIP, Tau, ATP
UBCH5B
UEV1 UBCH13
Time
5' 60' 5' 60'
C
UBCH5B 1st repeat
UBCH5B 2nd repeat
UEV1:UBCH13 1st repeat
UEV1:UBCH13 2nd repeat
Intensity
1200
800
400
0
600
400
200
30
15
20
10
Acidic Domain
Proline Rich Domain
Microtubule Binding Domain
C-terminal Domain
24 44 67 87 130 132 140 141 143 148 150 163 174 180 190 224 225 234 240 254 257 259 267 274 280 281 290 294 298 311 317 321 331 340 343 347 353 369 370 375 383 385 396 438
Lysine position
D
Ubiquitinated Tau
Fluorescent cargo receptor
Imaging
anti-FLAG beads
Tau coated beads
Tau - cargo receptor interaction

Fig. S1. Chaperone-dependent ubiquitylation renders Tau a selective autophagy cargo: additional data, biological repeats and experimental scheme.

- **A.** Biological repeats of Fig. 1A.
- **B.** Western Blot anti FLAG of Tau ubiquitylated in the absence of HSPA1A.
- **C.** Quantitative comparison of MS analysis repeats of Fig. 1D. Ubiquitin site intensities of Tau normalized to the protein intensity. Black bars: sites identified in orange regions in Fig. 1C; Grey bars: sites from blue and purple regions pooled.
- **D.** Scheme of a microscopy-based protein-protein interaction assay.

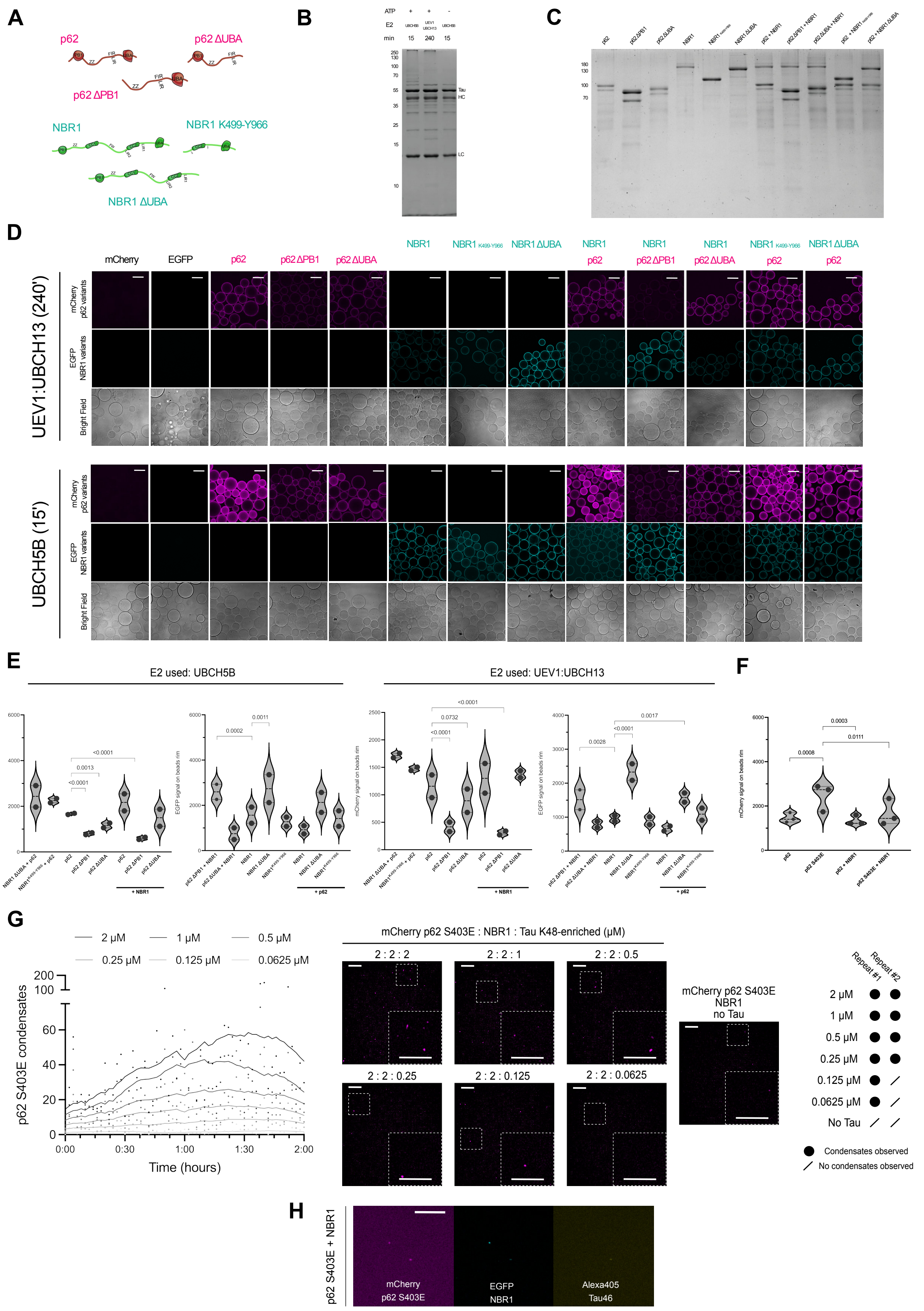
A
p62
p62 ΔUBA
p62 ΔPB1
NBR1
NBR1 K499-Y966
NBR1 ΔUBA
B
ATP
E2
min
C
D
mCherry
EGFP
p62
p62 ΔPB1
p62 ΔUBA
NBR1
NBR1 K499-Y966
NBR1 ΔUBA
UEV1:UBCH13 (240')
UBCH5B (15')
mCherry p62 variants
EGFP NBR1 variants
Bright Field
E
E2 used: UBCH5B
E2 used: UEV1:UBCH13
F
G
p62 S403E condensates
Time (hours)
mCherry p62 S403E : NBR1 : Tau K48-enriched (μM)
mCherry p62 S403E NBR1 no Tau
Condensates observed
No condensates observed
H
p62 S403E + NBR1
mCherry p62 S403E
EGFP NBR1
Alexa405 Tau46

Fig. S2. Mechanism of monomeric ubiquitylated Tau recognition by p62: NBR1 – biological repeats and controls

- **A.** Cartoon of p62 and NBR1 truncations used in this study.
- **B.** SDS PAGE of input ubiquitylated Tau. HC and LC = heavy and light chains of anti-FLAG antibody.
- **C.** SDS PAGE of prey levels.
- **D.** Microscopy-based protein-protein interaction assay as in Fig1E. Autophagy cargo receptors p62 and NBR1 are either full length or truncated. Scale bar 100 μm.
- **E.** Quantification of both UBCH5B and UEV1:UBCH13 conditions from Fig. S2D. A nested ANOVA was used to estimate significance.
- **F.** Binding of p62 variants (2 uM), +/- NBR1 (1 uM) to K63-enriched Tau on beads, quantified as in Fig. S2E. A nested ANOVA was used to estimate significance.
- **G.** Right: number of mCherry p62 (2 μm) condensates plus NBR1 (1 μm) over time at increasing concentrations of Tau K48-enriched. Lines indicate moving averages of 20 values. Middle: representative images (1.5 h of condensation), visualised by spinning disk microscopy. Scale bar 50 μm. Left: condensation thresholds for two independent repeats.
- **H.** Related to Fig. 2F, mock co-localisation of P62 S403E (2 uM), EGFP NBR1 (1 uM) and Alexa405:Tau46, assessed by confocal microscopy. Scale bar 100 μm.

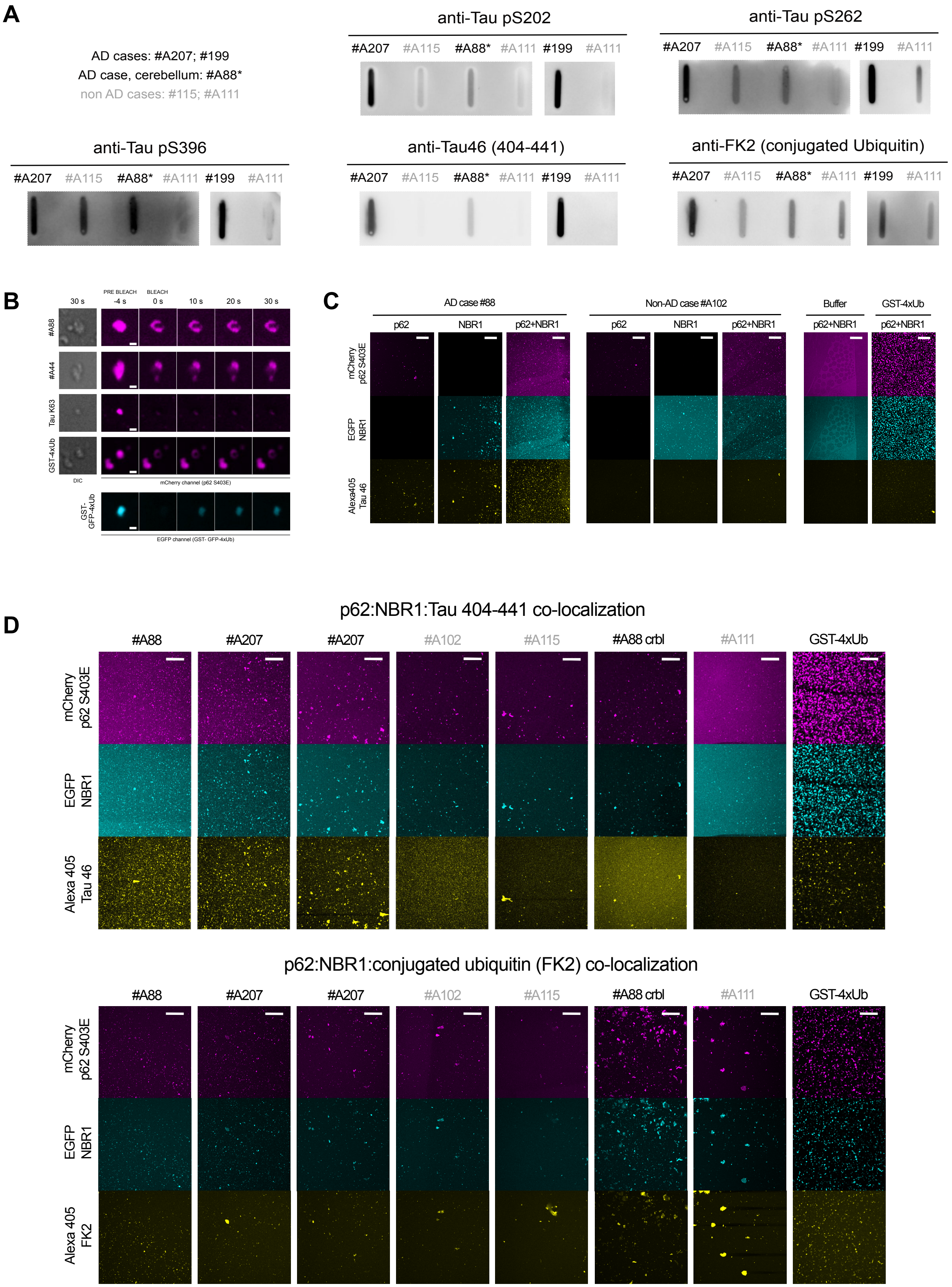
A
AD cases: #A207; #199
AD case, cerebellum: #A88*
non AD cases: #115; #A111
anti-Tau pS202
#A207 #A115 #A88* #A111 #199 #A111
anti-Tau pS262
#A207 #A115 #A88* #A111 #199 #A111
anti-Tau pS396
#A207 #A115 #A88* #A111 #199 #A111
anti-Tau46 (404-441)
#A207 #A115 #A88* #A111 #199 #A111
anti-FK2 (conjugated Ubiquitin)
#A207 #A115 #A88* #A111 #199 #A111
B
30 s
PRE BLEACH -4 s
BLEACH 0 s
10 s
20 s
30 s
#A88
#A44
Tau K63
GST-4xUb
DIC
mCherry channel (p62 S403E)
GST-GFP-4xUb
EGFP channel (GST- GFP-4xUb)
C
AD case #88
Non-AD case #A102
Buffer
GST-4xUb
p62
NBR1
p62+NBR1
mCherry p62 S403E
EGFP NBR1
Alexa405 Tau 46
D
p62:NBR1:Tau 404-441 co-localization
#A88 #A207 #A207 #A102 #A115 #A88 crbl #A111 GST-4xUb
mCherry p62 S403E
EGFP NBR1
Alexa 405 Tau 46
p62:NBR1:conjugated ubiquitin (FK2) co-localization
#A88 #A207 #A207 #A102 #A115 #A88 crbl #A111 GST-4xUb
mCherry p62 S403E
EGFP NBR1
Alexa 405 FK2

Fig. S3. Tau fibrils from *post-mortem* human brains characterisation – additional data

- **A.** Slot blot antibody characterisation of *post-mortem* sarkosyl insoluble fraction of both non-AD and AD cases.
- **B.** Fluorescent Recovery After Photobleaching of mCherry P62 S403E (2 uM) and NBR1 (1 uM) condensates in the presence of i) monomeric ubiquitylated Tau K63-enriched (4 h of condensation), ii) GST-4xUb (0.5 h of condensation), iii) two AD brain sections (frontal, 2 h of condensation) and iv) GST-GFP-4xUb (0.5 h of condensation), visualised by spinning disk microscopy. Scale bar 2 μm.
- **C.** Co-localisation of P62 S403E 2 uM and EGFP NBR1 1 uM – in combination or alone – with sarkosyl-insoluble fractions of non-AD and AD cases, assessed by confocal microscopy via Alexa405:Tau46. Scale bar 100 μm.
- **D.** Top panel: co-localisation of P62 S403E 2 uM and EGFP NBR1 1 uM with Tau fibrils from three AD and three non-AD cases, assessed by confocal microscopy via Alexa405:Tau46. Bottom panel: co-localisation of P62 S403E 2 uM and EGFP NBR1 1 uM with conjugated ubiquitin for three AD and three non-AD cases, assessed by confocal microscopy via Alexa405:FK2. Scale bar 100 μm.

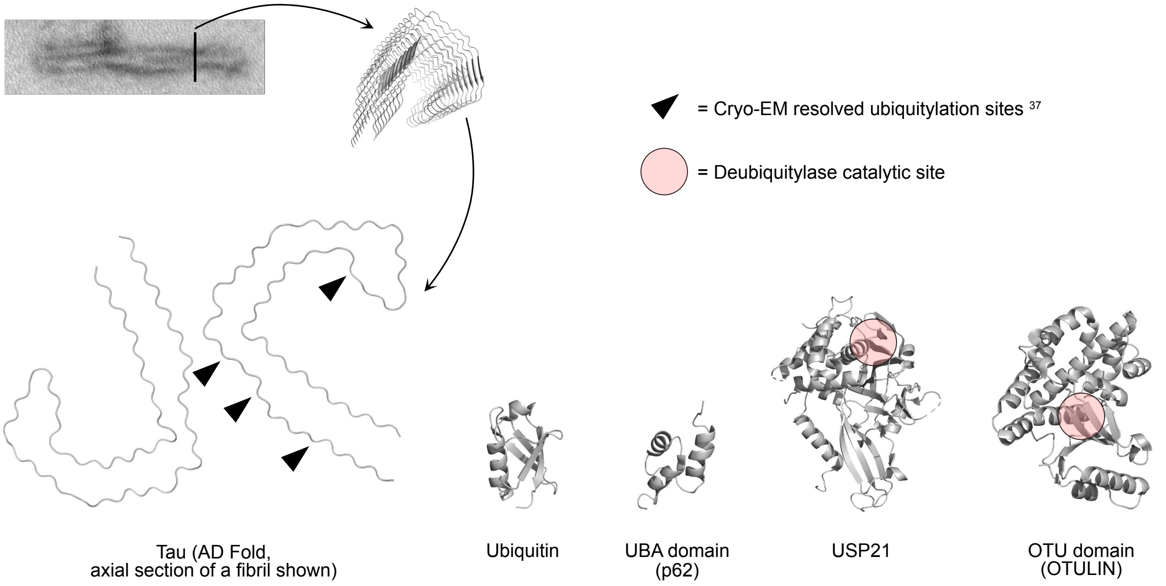

Fig. S4. Availability of Ubiquitin on Tau fibrils to p62 UBA domain and deubiquitylases
Tau fold from AD brains to scale with Ubiquitin (PDB file: 2KHW), the UBA domain of p62 (2JY7), USP21 (2Y5B) and the OTU domain of OTULIN (3ZNV). For clarity only one pair of protomers of a fibril is shown as top view. Pink circles indicate deubiquitylases catalytic sites. Arrows indicate ubiquitylated lysines associated to disease, as reported in [37].

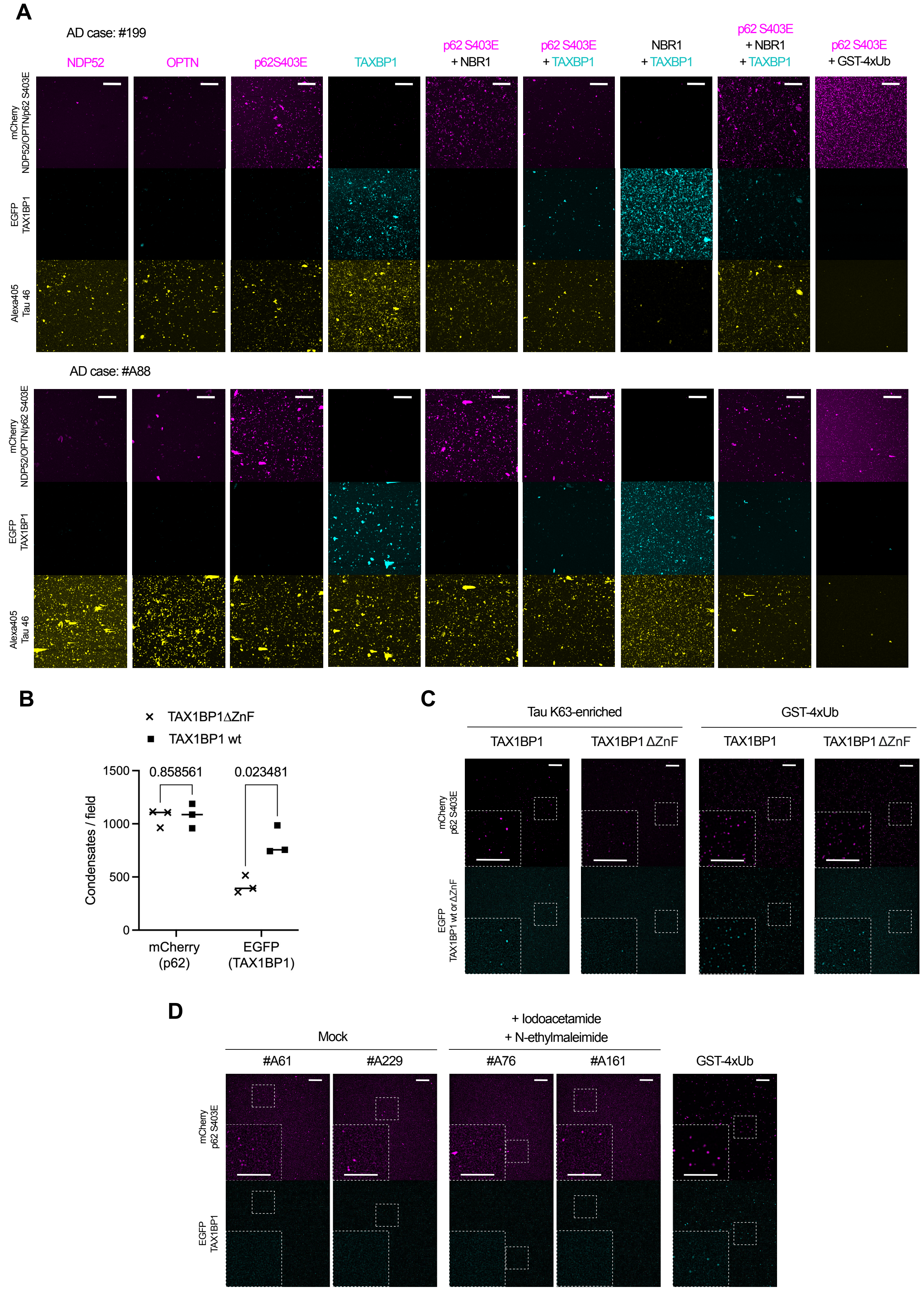
A
AD case: #199
NDP52
OPTN
p62S403E
TAXBP1
p62 S403E + NBR1
p62 S403E + TAXBP1
NBR1 + TAXBP1
p62 S403E + NBR1 + TAXBP1
p62 S403E + GST-4xUb
mCherry NDP52/OPTN/p62 S403E
EGFP TAX1BP1
Alexa405 Tau 46
AD case: #A88
B
TAX1BP1ΔZnF
TAX1BP1 wt
Condensates / field
0.858561
0.023481
1500
1000
500
0
mCherry (p62)
EGFP (TAX1BP1)
C
Tau K63-enriched
GST-4xUb
TAX1BP1
TAX1BP1 ΔZnF
mCherry p62 S403E
EGFP TAX1BP1 wt or ΔZnF
D
Mock
+ Iodoacetamide + N-ethylmaleimide
#A61
#A229
#A76
#A161
GST-4xUb
mCherry p62 S403E
EGFP TAX1BP1

Fig. S5. TAX1BP1 is excluded from pathological aggrephagy condensates – additional data

- **A.** Two biological repeats of Fig. 5B.
- **B.** Kinetics of condensate formation in the presence of mCherry P62 S403E (2 uM), NBR1 (1 uM), EGFP TAX1BP1 (either wt or ΔZnF) and GST-4xUb, both green and red channels imaged via spinning disk microscopy after 10 min of condensation. An unpaired, two-tailed Student's t-Test was used to estimate significance.
- **C.** Representative images of mCherry P62 S403E (2 uM), NBR1 (1 uM) and EGFP TAX1BP1 (either wt or ΔZnF) condensates in the presence of: i) monomeric ubiquitylated Tau K63-enriched (1 h of condensation) and ii) GST-4xUb (10 min of condensation), visualised by spinning disk microscopy. Scale bar 50 μm.
- **D.** Representative images of mCherry P62 S403E (2 uM) and EGFP TAX1BP1 (2 uM) condensates in the presence of i) fibrillar Tau purified from four AD brain sections (#A61 and #A76 *cornu ammonis*, hippocampus; #A161 and #A229 frontal, 1 h of condensation) and ii) GST-4xUb (1 h of condensation) visualised by spinning disk microscopy. Cases #A76 and #A161 were purified in the presence of the DUB inhibitors Iodoacetamide and N-ethylmaleimide. Scale bar 50 μm.

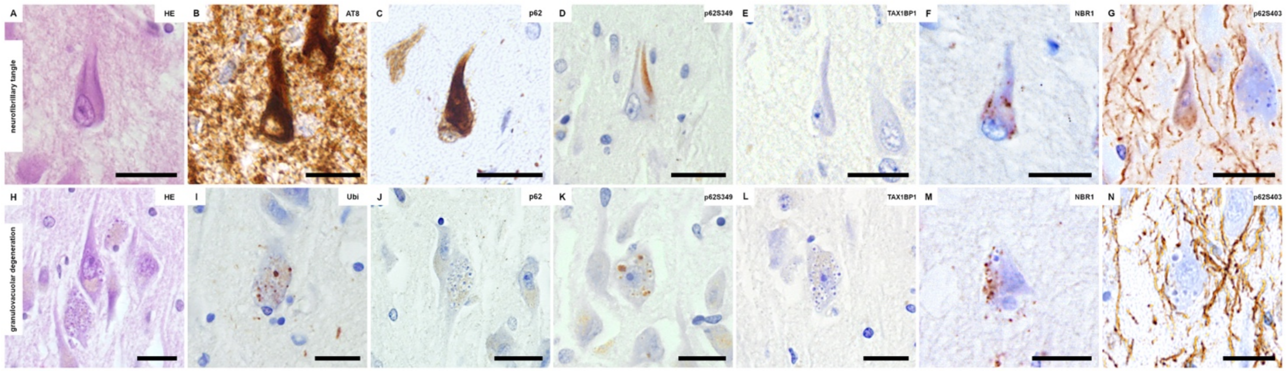

Fig. S6. Tau fibrils and autophagy cargo receptors p62 and NBR1 co-cluster in human brains with AD, while TAX1BP1 cannot be detected

A, B, C, H, I, J: Typical histological and immunohistochemical findings in an autopsy case of a patient with AD using different conventional antibodies used in standard neuropathological assessment. **A, B, C:** Histological and immunohistochemical representation of a neurofibrillary tangle using H&E staining (A, HE), immunohistochemical staining against hyperphosphorylated Tau showing strong staining of a neurofibrillary tangle (B, AT8) and p62 showing strong staining of a neurofibrillary tangle (C, p62). **H, I, J:** Histological and immunohistochemical representation of granulovacuolar degeneration using H&E staining (H, HE), immunohistochemical staining against Ubiquitin showing strong staining of the granular component (I, Ubi) and p62 lacking immunohistochemical staining of the granular component (J, p62). Scale bars: 20 µm for A, B, H, I; 25 µm for C, J.

D, E, F, G, K, L, M, N: Immunohistochemical findings in the autopsy case of a patient with AD using different antibodies related to autophagy. **D, E, F, G:** Immunohistochemical representation of a neurofibrillary tangle using an anti-p62 S349 antibody showing distinct staining of the neurofibrillary tangle (D, p62S349), anti-TAX1BP1 antibody lacking immunohistochemical staining (E, TAX1BP1), anti-NBR1 antibody showing a granular „lysosomal pattern" in neuronal and glial cells without staining of neurofibrillary tangles (F, NBR1), anti-p62 S403 antibody showing a strong staining of glial intermediate filaments and a less pronounced staining of a neurofibrillary tangle (G, p62S403). **K, L, M, N:** Immunohistochemical representation of granulovacuolar degeneration using an anti-p62 S349 antibody showing marked staining of the granular component (K, p62S349), anti-TAX1BP1 antibody lacking immunohistochemical staining (L, TAX1BP1), anti-NBR1 antibody showing a granular „lysosomal pattern" in neuronal cells without staining of the granular component (M, NBR1), anti-p62 S403 antibody showing a strong staining of glial intermediate filaments and a lack of staining of the granular component (N, p62S403). Scale bars: 20 µm for E, F, K, L, M, N; 25 µm for D, G.
